# Salvage of ribose from uridine or RNA supports glycolysis in nutrient-limited conditions

**DOI:** 10.1038/s42255-023-00774-2

**Published:** 2023-05-17

**Authors:** Owen S. Skinner, Joan Blanco-Fernández, Russell P. Goodman, Akinori Kawakami, Hongying Shen, Lajos V. Kemény, Lena Joesch-Cohen, Matthew G. Rees, Jennifer A. Roth, David E. Fisher, Vamsi K. Mootha, Alexis A. Jourdain

**Affiliations:** 1 Broad Institute of MIT and Harvard, Cambridge, MA USA; 2 Department of Molecular Biology and Howard Hughes Medical Institute, Massachusetts General Hospital, Boston, MA USA; 3 Department of Systems Biology, Harvard Medical School, Boston, MA USA; 4 Department of Immunobiology, University of Lausanne, Epalinges, Switzerland; 5 Cutaneous Biology Research Center, Department of Dermatology, Massachusetts General Hospital, Harvard Medical School, Charlestown, MA USA; 6 Department of Dermatology, Venereology and Dermatooncology, Faculty of Medicine, Semmelweis University, Budapest, Hungary; 7 Present Address: Liver Center, Division of Gastroenterology, Massachusetts General Hospital, Boston, MA USA; 8 Present Address: Cellular and Molecular Physiology, Yale School of Medicine, New Haven, CT USA; 9 Present Address: Yale Systems Biology Institute, Yale West Campus, West Haven, CT USA

**Keywords:** Metabolomics, Carbohydrates, Metabolism

## Abstract

Glucose is vital for life, serving as both a source of energy and carbon building block for growth. When glucose is limiting, alternative nutrients must be harnessed. To identify mechanisms by which cells can tolerate complete loss of glucose, we performed nutrient-sensitized genome-wide genetic screens and a PRISM growth assay across 482 cancer cell lines. We report that catabolism of uridine from the medium enables the growth of cells in the complete absence of glucose. While previous studies have shown that uridine can be salvaged to support pyrimidine synthesis in the setting of mitochondrial oxidative phosphorylation deficiency^1^, our work demonstrates that the ribose moiety of uridine or RNA can be salvaged to fulfil energy requirements via a pathway based on: (1) the phosphorylytic cleavage of uridine by uridine phosphorylase UPP1/UPP2 into uracil and ribose-1-phosphate (R1P), (2) the conversion of uridine-derived R1P into fructose-6-P and glyceraldehyde-3-P by the non-oxidative branch of the pentose phosphate pathway and (3) their glycolytic utilization to fuel ATP production, biosynthesis and gluconeogenesis. Capacity for glycolysis from uridine-derived ribose appears widespread, and we confirm its activity in cancer lineages, primary macrophages and mice in vivo. An interesting property of this pathway is that R1P enters downstream of the initial, highly regulated steps of glucose transport and upper glycolysis. We anticipate that ‘uridine bypass’ of upper glycolysis could be important in the context of disease and even exploited for therapeutic purposes.

## Abstract (web summary)

In this study, Skinner, Blanco-Fernández et al. show that uridine can be salvaged through the non-oxidative branch of the pentose phosphate pathway to feed glycolysis in conditions of glucose scarcity.

## Main

**Fig. 1:**
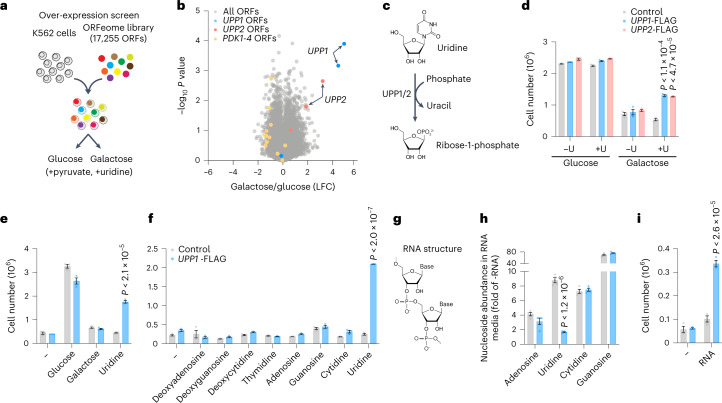
Uridine phosphorylase activity supports growth on uridine or RNA. **a**, Schematic overview of the ORF proliferation screen. **b**, Volcano plot representation of the screen hits after 21 d of growth in medium containing 25 mM glucose or 25 mM galactose, 0.2 mM uridine and 1 mM sodium pyruvate (*n* = 2). LFC, log_2_ (fold change). *P* values were calculated using a two-sided Student’s *t*-test. Statistics were not adjusted for multiple comparisons. **c**, Reaction catalysed by UPP1 and UPP2 proteins. **d**–**f**, Cell growth assays of K562 control cells and K562 cells expressing *UPP1*-FLAG or *UPP2*-FLAG in pyruvate-free media in the presence of: 25 mM glucose or 25 mM galactose or 0.2 mM uridine (±U; *n* = 3 replicate wells, *P* < 1.1 × 10^−4^ and *P* < 4.7 × 10^−5^; **d**), 10 mM of either glucose, galactose or uridine (*n* = 3, *P* < 2.1 × 10^−5^; **e**) or 5 mM of the indicated nucleosides (*n* = 3, *P* < 2.0 × 10^−7^; **f**). Data are shown as the mean ± s.e.m. with two-sided *t*-test relative to control cells. **g**, Schematic of RNA highlighting its ribose groups. **h**, Intracellular abundance of the four nucleoside precursors of RNA in control or *UPP1*-FLAG-expressing K562 cells grown in sugar-free medium supplemented with 0.5 mg ml^−1^ purified yeast RNA after 24 h. Data are expressed as fold changes of sugar-free medium (*n* = 4, *P* < 1.2 × 10^−6^) and shown as the mean ± s.e.m. with two-sided *t*-test relative to control. **i**, Cell growth assays of control or *UPP1*-FLAG-expressing K562 cells in sugar-free medium supplemented with 0.5 mg ml^−1^ of purified yeast RNA (*n* = 3, *P* < 2.6 × 10^−5^). Data are shown as the mean ± s.e.m. with two-sided *t*-test relative to control cells. All growth assays, metabolomics and screens included 4 mM l-glutamine and 10% dialysed FBS. Source data

**Extended Data Fig. 1:**
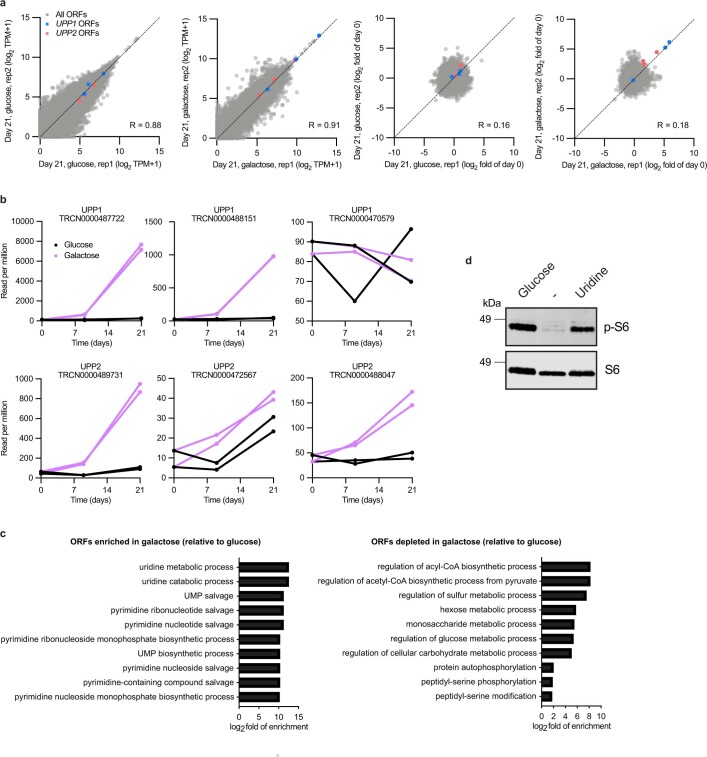
Additional analysis of the ORF screen. (**a**) Replicate plot and Pearson correlation (R) of *n* = 2 replicate ORF screens in glucose and galactose highlighting *UPP1* and *UPP2* ORFs shown as log_2_ TPM + 1, or log_2_ fold of day 0. (**b**) Representation of all six *UPP* ORFs, expressed as read per million in the global population of glucose or galactose-grown cells and as a function of time (*n* = 2). TRCN0000470579 encodes a splice variant of *UPP1* that is N-terminal truncated and lacks the uridine binding site (NM_001287428.2). (**c**) Top 10 ontologies associated with the ORFs enriched and depleted in galactose relative to glucose. The complete gene ontology analysis is reported in the Supplementary Data Table 1. (**d**) Protein immunoblot of K562 cells expressing *UPP1*-FLAG grown for 16 h in sugar-free media supplemented with 10 mM of glucose or uridine and immunolabelled with antibodies to total S6 ribosomal protein and phosphorylated S6 ribosomal protein (p-S6). Representative of *n* = 2 experiments. Total S6 loading control was performed on the same gel. All growth assays and screens included 4mM L-glutamine and 10% dialyzed FBS. Source data

We sought to identify new genes and pathways that might serve as alternative sources of energy when glucose is limiting. We transduced K562 cells with a library comprising 17,255 barcoded open reading frames (ORFs)^2^ and compared proliferation in medium containing glucose and galactose, a poor substrate for glycolysis (Fig. 1a). We used Dulbecco’s modified Eagle’s medium (DMEM) that contained glutamine, as well as pyruvate and uridine, for which oxidative phosphorylation (OXPHOS)-deficient cells are dependent^1,3^. After 21 d, we harvested cells and sequenced barcodes using next-generation sequencing (Extended Data Fig. 1a and Supplementary Table 1). The mitochondrial pyruvate dehydrogenase kinases 1–4 (encoded by *PDK1*–*PDK4*) are repressors of oxidative metabolism, and all four isoforms were depleted in galactose (Fig. 1b). Unexpectedly, we found striking enrichment in galactose for ORFs encoding UPP1 and UPP2, two paralogous uridine phosphorylases catalysing the phosphate-dependent catabolism of uridine into R1P and uracil (Fig. 1b,c and Extended Data Fig. 1b,c).

To validate the screen, we stably expressed *UPP1* and *UPP2* ORFs in K562 cells and observed a significant gain in proliferation in galactose medium (Fig. 1d). This gain was dependent on uridine being present in the medium, while expression of *UPP1/UPP2*, or addition of uridine, had no effect in glucose-containing medium. Importantly, we found that *UPP1*-expressing cells also efficiently proliferated in medium containing uridine in the complete absence of glucose or galactose (‘sugar-free’), while control cells were unable to proliferate (Fig. 1e and Extended Data Fig. 1d). The ability of *UPP1* cells to grow in sugar-free medium strictly depended on uridine, and none of the other seven nucleoside precursors of nucleic acids could substitute for uridine (Fig. 1f).

Uridine-derived nucleotides are building blocks for RNA (Fig. 1g), and RNA is an unstable molecule, sensitive to cellular and secreted RNases. We tested if RNA-derived uridine could support growth in a *UPP1*-dependent manner and supplemented glucose-free medium with purified yeast RNA. The intracellular abundance of all four ribonucleosides accumulated following addition of RNA to the medium, with significantly lower uridine levels in *UPP1*-expressing cells, suggesting *UPP1*-mediated catabolism (Fig. 1h). Accordingly, *UPP1*-expressing K562 cells proliferated in sugar-free medium supplemented with RNA (Fig. 1i). We conclude that elevated uridine phosphorylase activity confers the ability to grow in medium containing uridine or RNA, in the complete absence of glucose.

**Fig. 2:**
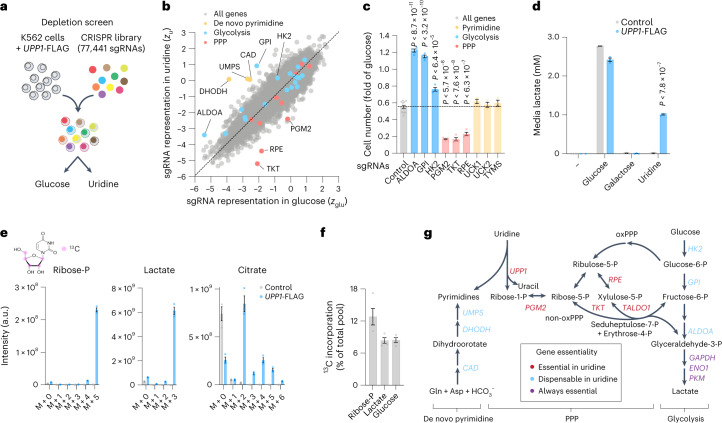
Uridine-derived ribose contributes to the pentose phosphate pathway and glycolysis. **a**, Schematic of a genome-wide CRISPR–Cas9 depletion screen comparing the proliferation of *UPP1*-FLAG-expressing K562 cells in sugar-free medium containing 10 mM glucose or uridine after 21 d (*n* = 2), in the absence of supplemental pyruvate and uridine. **b**, Gene-level analysis of a genome-wide CRISPR–Cas9 screen in glucose versus uridine reported as *z*-scores relative to non-cutting controls in glucose (*z*_glu_) and uridine (*z*_u_; *n* = 10,442 expressed genes, *n* = 2 replicates). **c**, Differential sensitivity of *UPP1*-FLAG-expressing K562 cells treated with the indicated sgRNAs targeting enzymes of upper glycolysis (*n* = 4, *P* < 8.7 × 10^−11^, *P* < 3.2 × 10^−10^, *P* < 6.4 × 10^−5^), the PPP (*n* = 4, *P* < 5.7 × 10^−8^, *P* < 7.6 × 10^−8^, *P* < 2.1 × 10^−5^, *P* < 6.3 × 10^−7^) or the salvage of uridine for pyrimidine synthesis (*n* = 3) in glucose versus uridine, expressed as the fold change of glucose and compared to control sgRNAs (*n* = 11). Data are shown as the mean ± s.e.m. after 4–5 d. *P* values were calculated using a two-sided Student’s *t*-test relative to control sgRNAs. Statistics were not adjusted for multiple comparisons. **d**, Lactate determination in medium containing 10 mM glucose, galactose or uridine (sugar-free) after 3 h (*n* = 3 replicate wells, *P* < 7.8 × 10^−7^). Data are shown as the mean ± s.e.m. with two-sided *t*-test relative to control cells. **e**, Labelling with ^13^C_5_-uridine ([1′,2′,3′,4′,5′-^13^C_5_]uridine; labelled carbon atoms in the ribose of uridine are indicated in magenta) and ^13^C_5_-uridine tracer analysis of representative intracellular metabolites from the PPP, glycolysis and the TCA cycle in control or *UPP1*-FLAG-expressing K562 cells (*n* = 3). Data are shown as the mean ± s.e.m. and are corrected for natural isotope abundance. **f**, ^13^C_5_-uridine tracer analysis of liver metabolites 30 min after intraperitoneal injection in overnight fasted mice with 0.4 g per kg body weight shown as the percentage of ^13^C-labelled intermediates compared to the total pool. Data are shown as the mean ± s.e.m. and are corrected for natural isotope abundance (*n* = 4 mice). **g**, Schematic of uridine-derived ribose catabolism integrating gene essentiality results in glucose versus uridine. Gln, glutamine; Asp, aspartate. All growth assays and metabolomics experiments included 4 mM (DMEM) l-glutamine and 10% dialysed FBS. a.u., arbitrary units. Source data

**Extended Data Fig. 2:**
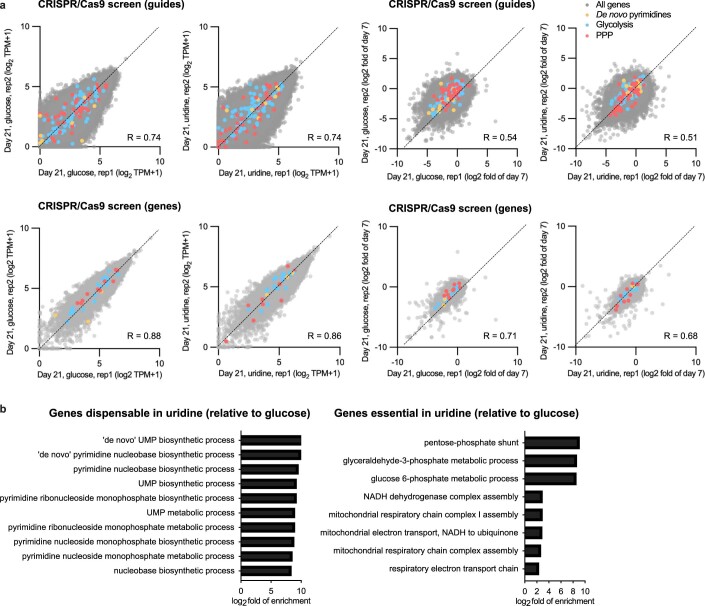
Genome-wide CRISPR–Cas9 screen and gene ontology analysis in glucose and uridine. (**a**) Replicate plot and Pearson correlation (R) analysis of *n* = 2 replicate genome-wide CRISPR–Cas9 screens in glucose and uridine highlighting genes of de novo pyrimidine synthesis, glycolysis and the PPP. Data are shown as log_2_ TPM + 1, or log_2_ fold of day 7. Top: Data shown at the individual sgRNA level. Bottom: Data shown at the gene level. (**b**) Top 10 ontologies associated with the genes enriched and depleted in uridine relative to glucose. Only 8 terms scored for the analysis of essential genes in uridine. The complete gene ontology analysis is reported in the Supplementary Data Table 1. Source data

We next addressed the mechanism of how uridine supports the growth of *UPP1*-expressing cells. Previous studies have noted the beneficial effect of uridine in the absence of glucose and proposed mechanisms that include the salvage of uridine for nucleotide synthesis and its role in glycosylation^4–8^. Others reported the beneficial role of uridine phosphorylase in maintaining ATP levels and viability during glucose restriction in the brain^9–11^. To further investigate the molecular mechanism of uridine-supported proliferation, we performed a secondary genome-wide CRISPR–Cas9 depletion screen using K562 cells expressing *UPP1*-FLAG grown on glucose or uridine (Fig. 2a,b and Extended Data Fig. 2a).

**Extended Data Fig. 3:**
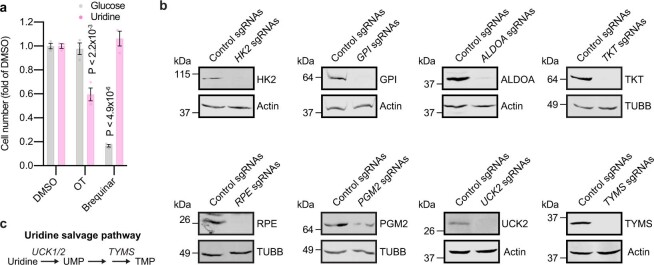
CRISPR–Cas9 screen validation. (**a**) Differential sensitivity to small molecule inhibitors of the PPP (OT: oxythiamine) or de novo pyrimidine synthesis (brequinar) in glucose *vs* uridine in *UPP1*-FLAG expressing K562 (*n* = 3, *P* < 2.2 × 10^−3^, *P* < 4.9 × 10^−6^) after 4 days, reported as fold of DMSO. Data are shown as ±SEM with two-sided t-test relative DMSO. (**b**) Immunoblot analysis of proteins from upper glycolysis, the PPP and pyrimidine salvage in *UPP1*-expressing K562 cells treated with their corresponding sgRNAs. *UCK1* is expressed at low levels in K562 cells and its protein could not be detected. Representative of *n* = 2 experiments. (**c**) Simplified representation of the uridine salvage pathway and thymidine synthesis. TUBB and Actin loading controls were performed on the same gels. All growth assays and screens included 4mM L-glutamine and 10% dialyzed FBS. Source data

We found that, although most essential gene sets were shared between glucose and uridine conditions, three major classes of genes were differentially essential in uridine as compared to glucose (Fig. 2b, Extended Data Fig. 2b and Supplementary Table 1): (1) As expected from pyrimidine salvage from uridine, all three enzymes involved in de novo pyrimidine synthesis (encoded by *CAD*, *DHODH* and *UMPS*) were essential in glucose but dispensable in uridine. (2) Genes central to the non-oxidative branch of the pentose phosphate pathway (non-oxPPP; *PGM2*, *TKT*, *RPE*) showed high essentiality in uridine. Among them *PGM2*, which encodes an enzyme that converts ribose-1-P to ribose-5-P and connects the UPP1/UPP2 reaction to the PPP, was highly essential in uridine, but almost fully dispensable in glucose. Accordingly, uridine-grown cells were particularly sensitive to depletion of *PGM2*, *TKT* and *RPE*, or to TKT inhibition, while they were insensitive to the de novo pyrimidine synthesis inhibitor brequinar(Fig. 2c and Extended Data Fig. 3a,b). In contrast, genes of the oxidative branch of the PPP (*G6PD*, *PGLS*, *PGD*) did not score differentially between glucose and uridine. (3) As expected from their essentiality in glucose-limited conditions^3,12^, genes encoding the mitochondrial respiratory chain were generally more essential in uridine, although to a lesser extent compared to the non-oxPPP, perhaps due to the low energy supply in the absence of glucose.

In contrast to the previously proposed mechanisms^4–8^, ablation of genes involved in uridine salvage for nucleotide synthesis (*UCK1*/UCK*2*, *TYMS*) or in glycosylation had no effect on the growth of cells in uridine when compared to glucose (Fig. 2c, Extended Data Fig. 3b,c and Supplementary Table 1). Central enzymes of glycolysis were essential both in glucose and in uridine, indicating that a functional glycolytic pathway is required for survival with uridine alone. However, our comparative analysis revealed that several upper glycolytic enzymes (encoded by *ALDOA*, *GPI* and *HK2*) were dispensable in uridine, and only essential in glucose (Fig. 2b,c and Extended Data Fig. 3b). Not all steps of upper glycolysis scored in either condition, potentially due to the multiple genes with overlapping functions encoding glycolytic enzymes, a common limitation in single gene-targeting screens. Nevertheless, genes found to be dispensable in uridine included all steps upstream of fructose-6-P (F6P) and/or glyceraldehyde-3-P (G3P), which connect the non-oxPPP to glycolysis, pointing to a key role for these two metabolites in supporting proliferation on uridine.

**Extended Data Fig. 4:**
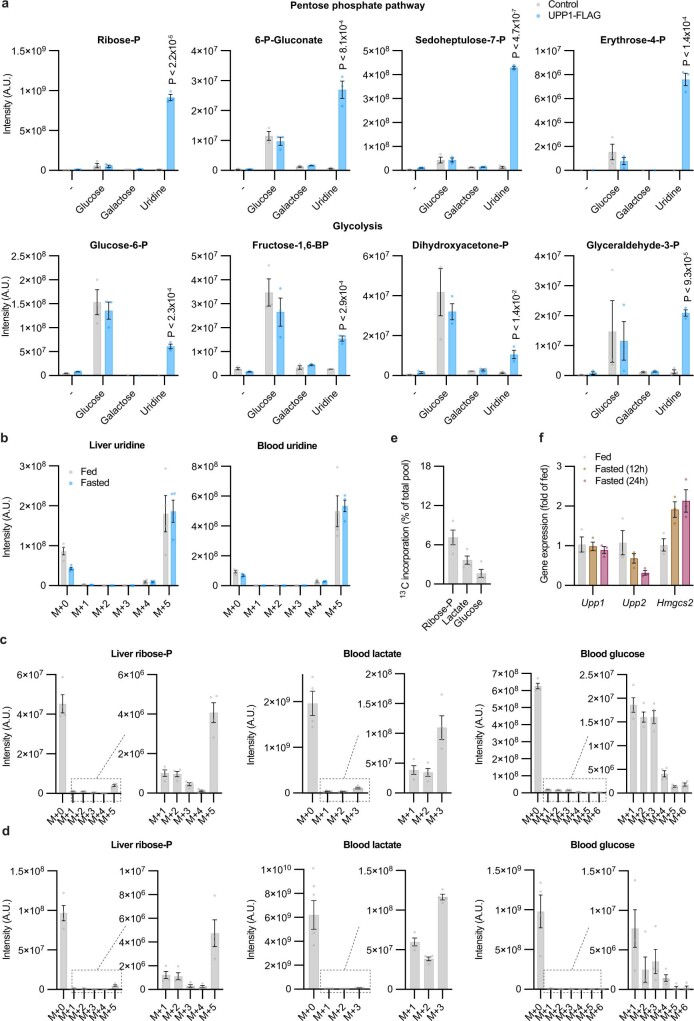
Additional metabolomics analysis. **(a)** Steady-state abundance of representative intracellular metabolites from the pentose phosphate pathway (PPP) and glycolysis in sugar-free media complemented with 10 mM glucose, 10 mM galactose or 10 mM uridine, in the presence of 4mM L-glutamine and 10% dialyzed FBS (*n* = 3 replicate wells, *P* < 2.2 × 10^−5^, *P* < 8.1 × 10^−4^, *P* < 4.7 × 10^−7^, *P* < 1.4 × 10^−4^). Data are shown as mean ± SEM with two-sided t-test relative to control cells. (**b**) ^13^C_5_-uridine tracer analysis of liver and blood uridine 30 min after intraperitoneal injection in fed or overnight fasted mice with 0.4 g/kg ^13^C_5_-uridine (*n* = 3 replicate wells, *P* < 2.3 × 10^−4^, *P* < 2.9 × 10^−4^, *P* < 1.4 × 10^−2^, *P* < 9.3 × 10^−5^). Data are shown as mean ± SEM (**c**) ^13^C_5_-uridine tracer analysis of liver ribose-phosphate (ribose-P) and circulating lactate and glucose 30 min after intraperitoneal injection in overnight fasted and (d) in fed animals with 0.4 g/kg ^13^C_5_-uridine. Data are shown as mean ± SEM and are corrected for natural isotope abundance (*n* = 4 mice in each group). (**e**) ^13^C_5_-uridine tracer analysis of liver ribose-phosphate, blood lactate and blood glucose 30 min after intraperitoneal injection in fed mice with 0.4 g/kg ^13^C_5_-uridine shown as the percentage of ^13^C-labeled intermediate compared to the total pool. Data are shown as mean ± SEM and are corrected for natural isotope abundance (*n* = 4 mice). See also (**f**) qPCR determination in the liver of ad libitum fed mice, or fasted for 12 h or 24 h, with probes to *Upp1*, *Upp2* and *Hmgc2*. *Hmgc2* transcripts are expected to increase with fasting. Data are shown as mean ± SEM (*n* = 3 mice in each group). Source data

The essentiality of the non-oxPPP, with the dispensability of upper glycolysis in uridine (Fig. 2b,c), prompted us to hypothesize that the ribose moiety of uridine can enter glycolysis and serve as a substrate for biosynthesis and energy production. Lactate secretion and glycolytic utilization of uridine, however, were excluded in earlier work^4–8^. Nonetheless, given the importance of PPP enzymes and the dispensability of upper glycolysis, we reinvestigated this possibility and measured lactate secretion in uridine-grown cells. Strikingly, we found that *UPP1*-expressing cells grown in uridine secreted high amounts of lactate (Fig. 2d). Accordingly, we found using liquid chromatography–mass spectrometry (LC–MS) that uridine restored steady-state abundance of most central carbon metabolism detected in the absence of glucose, strongly suggesting some degree of lower glycolysis activity from uridine (Extended Data Fig. 4a).

To directly test if uridine-derived ribose could serve as a substrate for glycolysis, we designed a tracer experiment using isotopically labelled uridine with five ribose carbons (^13^C_5_-uridine) and LC–MS (Fig. 2e). *UPP1*-expressing cells avidly incorporated ^13^C_5_-uridine, as seen by the presence of ^13^C in all the intracellular intermediates of the PPP and glycolysis analysed, including ribose-phosphate, upper and lower glycolytic intermediates and lactate, while control cells showed very little label incorporation. Tricarboxylic acid (TCA) cycle intermediates, among them citrate, were also partially labelled (mostly M + 2), indicating potential incorporation of carbon from glycolysis via pyruvate. To determine whether this labelling pattern extends in vivo, we next injected overnight fasted mice intraperitoneally with a ^13^C_5_-uridine tracer and measured incorporation in the liver and in circulating metabolites after 30 min. As in cell lines, we found ^13^C incorporation in ribose-phosphate and glycolysis in ^13^C_5_-uridine-treated animals (Fig. 2f and Extended Data Fig. 4b–e). Incorporation efficiency was smaller than in cell culture, as expected from low-dose ^13^C_5_-uridine injection, shorter treatment time and competition with other endogenous substrates in vivo, including unlabelled uridine. ^13^C_5_-uridine incorporation also occurred in fed animals, albeit to a lesser extent, and expression of liver *Upp1* and *Upp2* did not change with feeding (Extended Data Fig. 4b–f). We also found modest but significant incorporation of uridine-derived ^13^C in glucose, indicating gluconeogenesis from uridine-derived carbons (Fig. 2f and Extended Data Fig. 4c,d). Together, our results indicate that in cell lines and in animals in vivo, uridine catabolism provides ribose for the PPP, and that the non-oxPPP and the glycolytic pathway communicate via F6P and G3P to replenish glycolysis thus entirely bypassing the requirement for glucose in supporting lower glycolysis, biosynthesis and energy production in sugar-free medium (Fig. 2g).

**Fig. 3:**
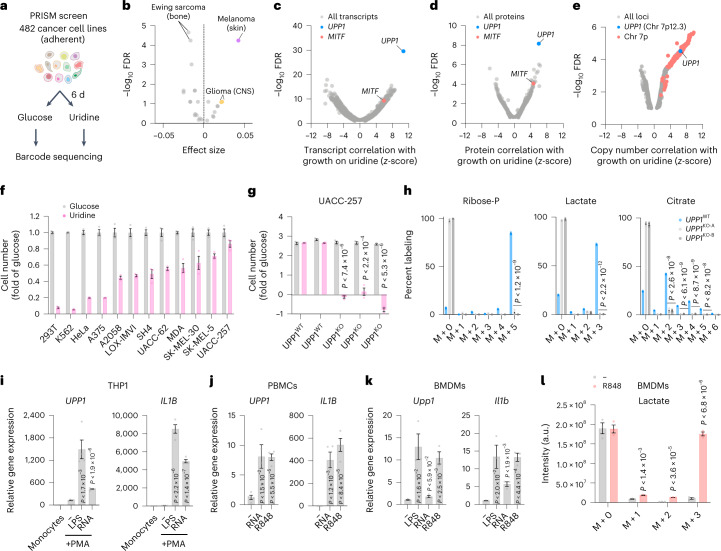
Capacity for glycolysis from uridine is governed by lineage and transcriptional control of *UPP1/UPP2* gene expression. **a**, Schematic of the PRISM screen with 482 cancer cells lines grown for 6 d in sugar-free medium complemented with 10 mM glucose or uridine (*n* = 2). **b**, Lineage analysis (*n* = 22 lineages) highlighting growth on uridine as compared to glucose. False discovery rates (FDRs) were calculated using a Benjamini–Hochberg algorithm correcting for multiple comparisons^13^. **c**,**d**, Correlation between uridine growth and expression of transcripts (*n* = 8,123; **c**) and proteins (*n* = 3,216; **d**) across cancer cell lines. **e**, Correlation between gene copy number (*n* = 5,950) and growth on uridine across the cell lines, highlighting chromosome 7p. *UPP1* is encoded on Chr7p12.3. **f**, Cell growth assay in sugar-free medium complemented with 10 mM glucose or uridine of a panel of melanoma (*n* = 9) and non-melanoma (*n* = 3, 293T, K562 and HeLa) cell lines. Data are shown as the mean ± s.e.m. (*n* = 4). MDA, MDA-MB-435S. **g**, Cell growth assay of melanoma UACC-257 wild-type (*UPP1*^WT^) and knock-out (*UPP1*^KO^) clones in sugar-free medium complemented with 10 mM of glucose or uridine. Data are shown as the mean ± s.e.m. (*n* = 3, *P* < 7.4 × 10^−6^, *P* < 2.2 × 10^−4^, *P* < 5.3 × 10^−6^) with two-sided *t*-test relative to *UPP1*^WT^ cells in the same medium. **h**, ^13^C_5_-uridine tracer analysis reporting representative intracellular metabolites from the PPP, glycolysis and the TCA cycle in UACC-257 wild-type (*UPP1*^WT^) and two knock-out (*UPP1*^KO^) clones after 5 h (*n* = 4, *P* < 1.2 × 10^−9^, *P* < 2.2 × 10^−12^, *P* < 2.6 × 10^−8^, *P* < 6.1 × 10^−9^, *P* < 8.7 × 10^−9^, *P* < 8.2 × 10^−8^). **i**–**k**, Expression of *UPP1* (*Upp1*) and *IL1B* (*Il1b*) in human THP1 cells (*n* = 4, *P* < 1.7 × 10^−3^, *P* < 1.9 × 10^−6^, *P* < 2.2 × 10^−6^, *P* < 1.4 × 10^−7^; **i**), human M-CSF-matured PBMCs (*n* = 4 donors, *P* < 1.5 × 10^−2^, *P* < 5.5 × 10^−5^, *P* < 1.2 × 10^−3^, *P* < 8.4 × 10^−5^; **j**) and BMDMs (*n* = 3 mice, *P* < 1.6 × 10^−2^, *P* < 5.9 × 10^−2^, *P* < 2.5 × 10^−3^, *P* < 2.0 × 10^−2^, *P* < 1.9 × 10^−3^, *P* < 4.4 × 10^−4^; **k**) after treatment with 100 nM phorbol myristate acetate (PMA) for 48 h (THP1), 100 ng ml^−1^ lipopolysaccharides (LPS; THP1, BMDMs), 1 mg ml^−1^ purified yeast RNA (THP1, PBMCs, BMDMs) or 5 µg ml^−1^ of TLR7/TLR8 agonist (R848) for 24 h and as determined by quantitative PCR (qPCR). **l**, ^13^C_5_-uridine tracer analysis reporting incorporation in media lactate from BMDMs treated for 24 h with 5 µg ml^−1^ R848 and further grown for 16 h in glucose-free DMEM containing 5 mM ^13^C_5_-uridine and 5 µg ml^−1^ R848 (*n* = 3 mice, *P* < 1.4 × 10^−3^, *P* < 3.6 × 10^−5^, *P* < 6.8 × 10^−6^). Data are shown as the mean ± s.e.m. with two-sided *t*-test relative to untreated cells. Source data

**Extended Data Fig. 5:**
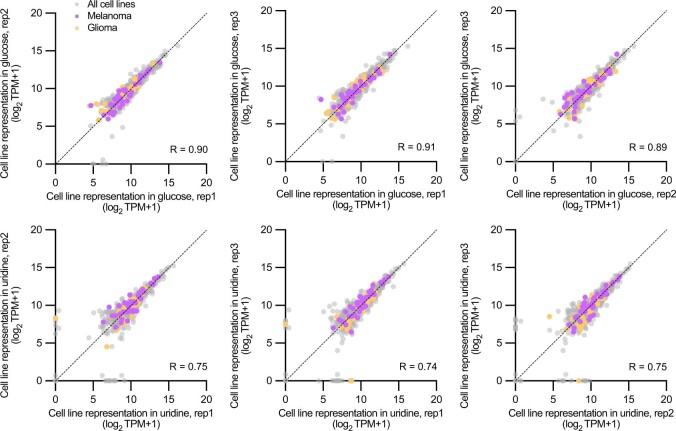
PRISM screen replicate analysis. Replicate plot and correlation analysis of *n* = 3 replicate PRISM screens in glucose and uridine highlighting the melanoma and glioma lineages and showing Pearson correlation between replicates (R). Source data

**Extended Data Fig. 6:**
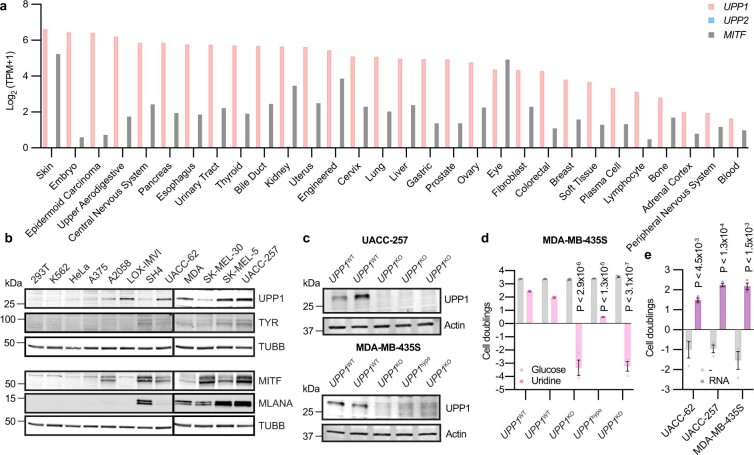
*UPP1*, *UPP2* and *MITF* expression across the CCLE collection and in melanoma. (**a**) *UPP1*, *UPP2* and *MITF* expression across the complete CCLE collection (*n* = 30 lineages). (**b**) Protein immunoblot of a panel of melanoma (*n* = 9) and non-melanoma (*n* = 3, 293T, K562 and HeLa) cell lines grown and showing expression of UPP1 as well as MITF, TYR and MLANA, three melanoma markers. MDA: MDA-MB-435S. (**c**) Top: Immunoblot of UACC-257 melanoma cells with wild-type (*UPP1*^WT^) and knock-out (*UPP1*^KO^). Bottom: Immunoblot of MDA-MB-435S melanoma cells in wild-type (*UPP1*^WT^), knock-out (*UPP1*^KO^) and hypomorphic (*UPP1*^hypo^) clones (see methods). (**d**) Cell growth assay of MDA-MB-435S clones in sugar-free media containing dialyzed FBS complemented with 10 mM of either glucose or uridine and dialyzed FBS. Negative doublings indicate cell death. Data are shown as mean ± SEM with two-sided t-test relative to *UPP1*^WT^ cells in the same media (*n* = 3 replicate wells, *P* < 2.9 × 10^−6^, *P* < 1.3 × 10^−5^, *P* < 3.1 × 10^−7^). (**e**) Cell growth assay of three melanoma cell lines with high *UPP1* expression in sugar-free media complemented with 10 mM of glucose or 0.5 mg/mL of RNA. Data are shown as mean ± SEM with two-sided t-test relative to *UPP1*^WT^ and were not corrected for multiple comparison (*n* = 3 replicate wells, *P* < 4.5 × 10^−3^, *P* < 1.3 × 10^−4^, *P* < 1.5 × 10^−3^. TUBB and Actin loading controls were performed on the same gels. All growth assays and screens included 4mM L-glutamine and 10% dialyzed FBS. Source data

We next sought to determine whether any human cell lines exhibit a latent ability to use uridine-derived ribose to grow on uridine when glucose is absent without the need for over-expression. We screened 482 pooled barcoded adherent cancer cell lines spanning 22 solid tumour lineages from the PRISM collection^13^ in medium containing 10 mM glucose or uridine, in the absence of any supplemental sugar (Fig. 3a, Extended Data Fig. 5 and Supplementary Table 1). Cells from the melanoma and the glioma lineages grew remarkably well in uridine as compared to the other lineages, whereas Ewing sarcoma cells grew significantly less well (Fig. 3b). Cell lines from the PRISM collection have been extensively characterized at a molecular level^14^, so we searched for genomic factors that correlate with the ability to grow on uridine (Supplementary Table 1). Genome wide, the top-scoring transcript, protein and genomic copy number variant was *UPP1* (Fig. 3c–e), in strong agreement with our ORF screen (Fig. 1b). Expression of *UPP1* across the CCLE collection was the highest in cell lines of skin origin (Extended Data Fig. 6a,b), where high uridine phosphorylase enzyme activity has been documented^15^, and tended to be lowest in the bone lineage. *UPP2* was almost never expressed in the CCLE collection (average transcripts per million (TPM) < 1; Extended Data Fig. 6a). In agreement with these results, we confirmed significant, *UPP1*-dependent, proliferation and uridine catabolism in melanoma cells grown in sugar-free medium supplemented with uridine or RNA (Fig. 3f–h and Extended Data Fig. 6c–e). We conclude that the endogenous expression of *UPP1* is necessary and sufficient to support the growth of cancer cells on uridine.

**Extended Data Fig. 7:**
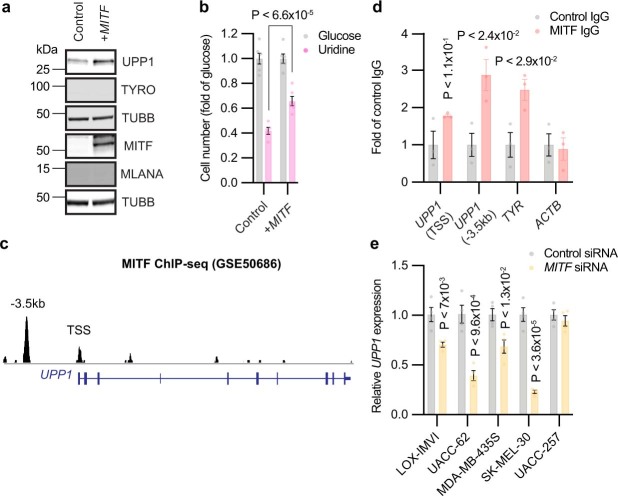
*MITF* promotes *UPP1* expression and growth on uridine in melanoma cells. (**a**) Protein immunoblot and (**b**) cell growth assay of LOX-IMVI in sugar-free media supplemented with 10 mM of glucose or 10 mM of uridine. LOX-IMVI is a melanoma cell line with low endogenous MITF expression and over-expressing *MITF* or a control gene. Data are shown as mean ± SEM (*n* = 3, *P* < 6.6 × 10^−5^). *P* values were calculated using a two-sided student T test. Statistics were not adjusted for multiple comparison. (**c**) MITF occupancy in *UPP1* transcription start site (TSS) and promoter (a region 3.5 kb away from the TSS), as determined by ChIP-Seq in COLO829 melanoma cells^16^. (**d**) ChIP-qPCR validation of MITF binding in *UPP1* promoter and TSS in MDA-MB-435S melanoma cells. *TYR* is a known transcriptional target of MITF, *ACTB* is not. Data are shown as mean ± SEM (*n* = 3, *P* < 1.1 × 10^−1^, *P* < 2.4 × 10^−2^, *P* < 2.9 × 10^−2^) with two-sided t-test relative to control IgG. (**e**) qPCR analysis of five melanoma cells after treatment with MITF siRNA (*n* = 3, *P* < 7.0 × 10^−3^, *P* < 9.6 × 10^−4^, *P* < 1.3 × 10^−2^, *P* < 3.6 × 10^−5^). Data are shown as mean ± SEM with two-sided t-test relative to the indicated control. TUBB loading controls were performed on the same gels. All growth assays and screens included 2 mM (RPMI) or 4 mM (DMEM) L-glutamine and 10% dialyzed FBS. Source data

We next investigated the factors that promote *UPP1* expression and growth on uridine by integrating our results with CCLE data to prioritize transcription factors, which highlighted *MITF* as a strong candidate in melanoma cells, both at the protein and the transcript level (Fig. 3c,d and Extended Data Fig. 6a,b). We found that *MITF* over-expression promoted *UPP1* expression and uridine growth (Extended Data Fig. 7a,b), while endogenous MITF binding was detected in the transcription start site (TSS) and the promoter (−3.5 kb from the TSS) of *UPP1* in a large-scale chromatin immunoprecipitation (ChIP) study^16^, which we experimentally validated (Extended Data Fig. 7c,d). Accordingly, siRNA-mediated depletion of *MITF* decreased *UPP1* expression in melanoma cells (Extended Data Fig. 7e).

**Extended Data Fig. 8:**
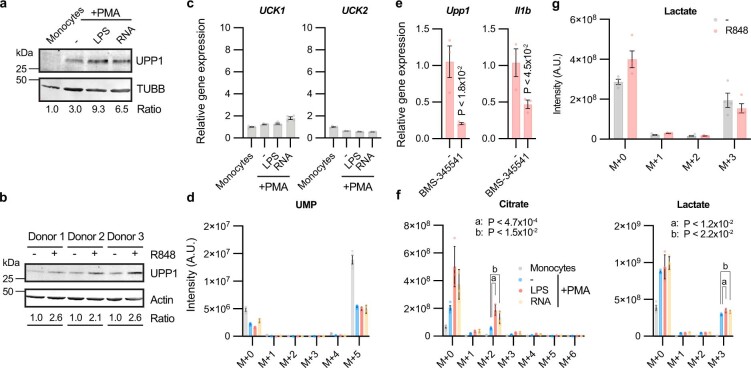
*UPP1* expression and uridine catabolism for energy production in the monocytic lineage. (**a**) Protein immunoblot of human THP1 monocytic cells treated with 100 nM PMA alone for 48 h followed by the addition of 100 ng/mL LPS or 1 mg/mL purified yeast RNA for another 48 h and immunoblotted with antibodies to UPP1 and TUBB. Western blot quantification is shown as fold of untreated cells (monocytes). (**b**) Protein immunoblot of human MCSF-matured PBMC treated with 5 µg/ml R848 for 24 h and immunoblotted with antibodies to UPP1 and Actin (*n* = 3 donors). Western blot quantification is shown as fold of untreated cells and relative to each donor. (**c**) Expression of *UCK1* and *UCK2* in THP1 cells as determined by qPCR after treatment as in (a). Data are shown as mean ± SEM with *n* = 4. (**d**) ^13^C_5_-uridine tracer analysis of UMP in THP1 cells treated as in (a) with the exception that glucose-free RPMI media containing 5 mM ^13^C_5_-uridine was used. Data are shown as mean ± SEM with *n* = 4. (**e**) Expression of *Upp1* and *Il1b* in BMDM as determined by qPCR after co-treatment for 24 h with 5 µg/ml R848 and 5 µg/ml BMS-345541, an IKK inhibitor. Data are shown as mean ± SEM with two-sided t-test relative to untreated (*n* = 3 mice, *P* < 1.8 × 10^−2^, *P* < 4.5 × 10^−2^). (**f**) ^13^C_5_-uridine tracer analysis of citrate and lactate in THP1 cells treated as in (a) with the exception that glucose-free RPMI media containing 5 mM ^13^C_5_-uridine was used for the last 6 h. Data are shown as mean ± SEM with two-sided t-test relative to PMA-treated cells with *n* = 4, *P* < 4.7 × 10^−4^, *P* < 1.5 × 10^−2^, *P* < 1.2 × 10^−2^, *P* < 2.2 × 10^−2^. (**g**) ^13^C_5_-uridine tracer analysis of media lactate in human MCSF-matured PBMC cells treated as in (b) with the exception that glucose-free RPMI media containing 5 mM ^13^C_5_-uridine was used was used for the last 6 h. Data are shown as mean ± SEM, with *n* = 4 donors. TUBB and Actin loading controls were performed on the same gels. All metabolomics included 2 mM (RPMI) or 4 mM (DMEM) L-glutamine and 10% dialyzed FBS. Source data

Our solid tumour PRISM cancer cells collection did not include cells of the immune lineage, where *UPP1* is expressed at high levels^17,18^, so we asked whether immune cells exhibit the capacity to metabolize ribose from uridine either at baseline or in a transcriptionally regulated manner. In the human monocytic THP1 cell line, in macrophage colony-stimulating factor (M-CSF)-matured peripheral blood mononuclear cells (PBMCs), and in primary mouse bone marrow-derived macrophages (BMDMs), we found that differentiation into macrophages and/or further polarization with immunostimulatory molecules increased *UPP1* expression (Fig. 3i–k and Extended Data Fig. 8a,b). In contrast, expression of pyrimidine salvage genes (*UCK1*/*UCK2*) and ^13^C_5_-uridine incorporation into UMP were not affected, and even decreased, during this process (Extended Data Fig. 8c,d). Among the immunostimulatory molecules, RNA enhanced *UPP1* expression, suggesting the existence of a feed-forward loop, where RNA (and conceivably RNA-containing pathogens and debris) may trigger *UPP1* expression and uridine salvage for building blocks and energy production. Supporting this idea, stimulation of PBMCs and BMDMs with a TLR7/TLR8 agonist (R848) lead to a significant, IκB kinase (IKK)-dependent, increase in *UPP1* transcription in BMDMs (Fig. 3j,k and Extended Data Fig. 8e). Label incorporation from uridine ribose was also strongly increased in citrate and lactate after differentiation of THP1 and after BMDM stimulation with R848, while it wasn't further increased in M-CSF-matured PBMCs, possibly due to high baseline capacity for uridine catabolism in these cells (Fig. 3l and Extended Data Fig. 8f,g). Together, our results indicate that macrophages have the capacity to use uridine-derived ribose for glycolysis, and that *UPP1* expression and uridine catabolism can sharply increase during cellular differentiation and in response to immunostimulating molecules, with cell type and species differences.

**Fig. 4:**
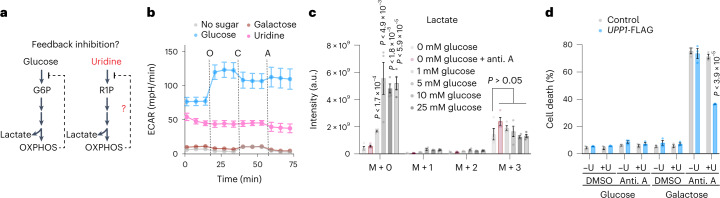
Glycolysis from uridine bypasses the regulated steps of upper glycolysis and supports OXPHOS-deficient cells. **a**, Schematic of glycolysis inhibition by OXPHOS. G6P, glucose-6-phosphate. **b**, Representative ECAR in *UPP1*-expressing K562 cells grown in sugar-free medium with and without supplementation of 10 mM of glucose, galactose or uridine, with *n* = 30 replicate wells. O, oligomycin; C, CCCP; A, antimycin A. Data are shown as the mean ± s.d. **c**, ^13^C_5_-uridine tracer analysis reporting intracellular lactate in UACC-257 melanoma cells in glucose-free RPMI medium containing 5 mM ^13^C_5_-uridine and in competition with increasing amount of unlabelled glucose (0, 1, 5, 10 and 25 mM) or treated with 100 nM antimycin A, all after 5 h (*n* = 4, *P* < 1.7 × 10^−4^, *P* < 4.9 × 10^−3^, *P* < 1.8 × 10^−5^, *P* < 5.9 × 10^−5^, *P* > 0.05). Data are shown as the mean ± s.e.m. and are corrected for natural isotope abundance. *P* values were calculated using a two-sided Student’s *t*-test. Statistics were not adjusted for multiple comparisons. **d**, Percentage of dead cells in *UPP1*-expressing K562 cells grown in 5 mM glucose or galactose supplemented with 5 mM uridine (U) and antimycin A (anti. A). Data are shown as the mean ± s.e.m. with two-sided *t*-test relative to control K562 cells (*n* = 4, *P* < 3.9 × 10^−5^). Source data

**Extended Data Fig. 9:**
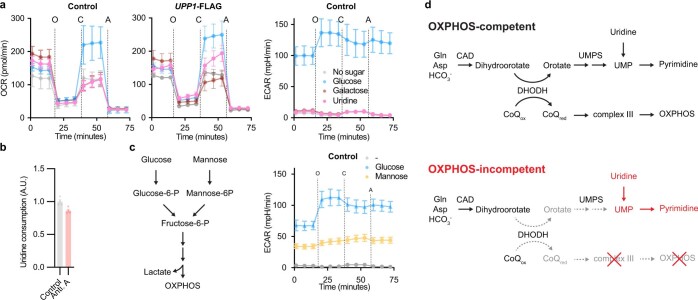
Additional bioenergetics measurement in uridine or mannose-grown cells. (**a**) Representative oxygen consumption rate (OCR) and extracellular acidification rate (ECAR) in control and *UPP1*-expressing K562 cells grown in sugar-free media or in media supplemented with 10 mM of glucose, galactose or uridine, all in the presence of glutamine and dialyzed FBS (*n* = 30 replicate wells). O: oligomycin. C: CCCP. A: antimycin A. Data are shown as mean ± SD with *n* > 6 replicate wells. (**b**) Total consumption of uridine in UACC-257 melanoma cells treated with antimycin A (100 nM) in the same media. Data are shown as mean ± SEM with *n* = 4. (**c**) Representative extracellular acidification rate (ECAR) in cells in 10 mM of glucose or mannose and treated as in (a). (**d**) Schematic representation of de novo pyrimidine synthesis and uridine auxotrophy during OXPHOS inhibition. CoQ: co-enzyme Q. Ox: oxidized. Red: reduced. All experiments included 4mM L-glutamine and 10% dialyzed FBS. Source data

We next sought to determine whether glycolysis from uridine is under acute regulation in the same way as from glucose. Active OXPHOS tends to keep glucose uptake and glycolysis at lower levels, while acute inhibition of OXPHOS leads to an immediate and strong increase in glucose-supported glycolysis, as evidenced by a robust increase in the extracellular acidification rate (ECAR) following oligomycin treatment (Fig. 4a,b). Strikingly, we found no ECAR stimulation by OXPHOS inhibitors, no difference in ^13^C_5_-uridine incorporation following antimycin blockage of the electron transport chain, and no increase in uridine import in OXPHOS-inhibited *UPP1*-expressing cells grown on uridine (Fig. 4b,c and Extended Data Fig. 9a,b). Because glycolysis from both uridine and glucose share a common pathway from G3P (Fig. 2g), differential regulation of glycolysis following OXPHOS inhibition must occur in the upper part of the pathway. Consistent with this notion, we observed no stimulation of ECAR in mannose-grown cells, a sugar connected to glycolysis by F6P (Extended Data Fig. 9c). We conclude that substrates such as uridine can enter glycolysis in a constitutive way, in contrast to glucose, by bypassing regulatory steps of upper glycolysis such as glucose transport and initial phosphorylation.

In line with this, we next performed a competition experiment to evaluate if the presence of glucose affects the incorporation of uridine in cells. Incorporation of uridine in lactate was notably not affected by competition with glucose in our experimental conditions, despite the presence of a large molar excess of glucose (Fig. 4c). Therefore, and in agreement with a bypass of regulatory steps of upper glycolysis, uridine can be incorporated into cells even when lactate production from glucose is saturated, suggesting constitutive import and catabolism.

Cells with severe OXPHOS dysfunction classically have to be grown on glucose, and uridine must be supplemented^1^. The traditional explanation has been that glucose is required to support glycolytic ATP production as OXPHOS is debilitated, and that uridine supplementation is required for pyrimidine salvage given that de novo pyrimidine synthesis via DHODH requires coupling to a functional electron transport chain^1,3^ (Extended Data Fig. 9d). Having observed energy harvesting from uridine, we finally tested whether uridine-derived ribose could also benefit OXPHOS-inhibited cells in the absence of glucose. We found a significant *UPP1*-dependent rescue of viability in galactose-grown cells treated with antimycin A (Fig. 4d), now revealing that supplemental uridine benefits mitochondrial dysfunction in two ways: (1) pyrimidine salvage when de novo pyrimidine synthesis is impossible, and (2) energy production in *UPP1*-expressing cells.

For decades it has been known that cells with mitochondrial deficiencies are dependent on uridine to support pyrimidine synthesis given the dependence of de novo pyrimidine synthesis on DHODH, whose activity is coupled to the electron transport chain^1^. Although it has been documented, it is less appreciated that uridine supplementation can support cell growth in the absence of glucose^4–10^. Here, we show that, in addition to nucleotide synthesis, uridine can serve as a substrate for energy production, biosynthesis and gluconeogenesis. Mechanistically, we show that glycolysis from uridine-derived ribose is initiated with the phosphorylytic cleavage of uridine by UPP1/UPP2, followed by shuttling of its ribose moiety through the non-oxPPP and glycolysis, hence supporting not only nucleotide metabolism but also energy production or gluconeogenesis in the absence of glucose (Fig. 2g).

By comparing uridine to other nucleosides and using similar tracer experiments to ours, Wice et al.^7^ observed incorporation of uridine-derived carbons in most cellular fractions in mammalian cell culture and in chicken embryos. However, they did not detect pyruvate and lactate in uridine, and concluded that uridine does not participate in glycolysis, but rather is required for nucleotide synthesis, and proposed that energy is derived exclusively from glutamine in the absence of glucose^6,7^. Loffler et al. and Linker et al. reached the same conclusion^4,8^. Our observations based on a genome-wide CRISPR–Cas9 screening and metabolic tracers (Fig. 2) agree with previous observations that cells can proliferate in sugar-free medium if uridine is provided, and that uridine is crucial for nucleotide synthesis—but differ mechanistically on the role of glycolysis in this condition, as we were able to identify a significant amount of labelling in glycolytic intermediates and secreted lactate, as well as a high ECAR, all consistent with glycolytic ATP production from uridine. It has previously been reported that uridine protects cortical neurons and immunostimulated astrocytes from glucose deprivation-induced cell death, in a way related to ATP, and it was hypothesized that uridine could serve as an ATP source^9^. Our genetic perturbation and tracer studies are consistent with this hypothesis.

The capacity to harvest energy and building blocks from uridine appears to be widespread. Here, we report very high capacity for uridine-derived ribose catabolism in melanoma and glioma cell lines (Fig. 3a–h), in primary human and mouse macrophages (Fig. 3i–l), and we also detect labelling patterns from uridine-derived ribose in the liver and the whole organism in vivo (Fig. 2f and Extended Data Fig. 4c–e). Our gain-of-function and loss-of-function studies suggest that tissues expressing *UPP1/UPP2* will have capacity for glycolysis from uridine-derived ribose. Based on gene expression atlases^18,19^, we predict uridine may be a meaningful source of energy in blood cells, lung, brain and kidney, as well as in certain cancers. Uridine is the most abundant and soluble nucleoside in circulation^20^ and it is possible that uridine may serve as an alternative energy source in these tissues, or for immune and cancer metabolism, similar to what has been proposed for other sugars and nucleosides^21–23^. It is notable that the strongest human metabolic quantitative trait loci for circulating uridine corresponds to *UPP1* (ref. ^24^), while uridine phosphorylase activity is the main determinant of circulating uridine in mice^25^.

A fascinating aspect of glycolysis from uridine is its apparent absence of regulation, at least at shorter timescales. The ability of uridine to serve as a constitutive input into glycolysis might have clinical implications for human diseases, as uridine is present at high levels in foods such as milk and beer^26,27^, and previous in vivo studies have shown that a uridine-rich diet leads to glycogen accumulation, gluconeogenesis, fatty liver and pre-diabetes in mice^28,29^. We now report that glycolysis from uridine lacks at least two checkpoints as (1) it is not controlled by OXPHOS (Fig. 4a–c), and (2) it occurs even when lactate production from glucose is evidently saturated (Fig. 4c), or after food intake in vivo (Extended Data Fig. 4d,e). Although glycolysis from uridine appears to occur at a slower pace than from glucose, we speculate that constitutive fuelling of glycolysis and gluconeogenesis from a uridine-rich diet may contribute to human conditions such as fatty liver disease and diabetes. Such a ‘uridine bypass’ is conceivable because glycolysis is so strongly controlled in upper glycolysis, for example, glucose transport^30^, which we show is bypassed by uridine, because the non-oxPPP and glycolysis are connected by F6P and G3P (Fig. 2g). This ability of uridine to bypass upper glycolysis may be beneficial in certain cases. For example, disorders of upper glycolysis, notably GLUT1 deficiency syndrome^31^, may benefit from uridine therapy and from induction of *UPP1/UPP2* expression.

At longer timescales, *UPP1* expression and capacity for ribose catabolism from uridine appear to be determined by cellular differentiation and further activation by extracellular signals. Here we focused on the monocytic lineage and found that (1) in THP1 cells, *UPP1* expression and activity sharply increased during differentiation and polarization, (2) high baseline rates of glycolysis from uridine are observed in M-CSF-matured PBMCs and (3) treatment with immunostimulating molecules acutely promote both *UPP1* expression and uridine catabolism in BMDMs (Fig. 3i–l). Whereas we didn’t investigate whether uridine is required for macrophage activation, we noticed that all the agonists tested ultimately lead to nuclear factor kappa B (NF-κB) activation, which binds in the *UPP1* promoter^17,32^. It is thus likely that NF-κB may serve as a transcription factor for *UPP1*. Supporting this assertion, we found that blocking NF-κB signalling with upstream IKK inhibitors abolished R848-induced *Upp1* expression (Extended Data Fig. 8e).

Uridine phosphorylase and ribose salvage by *UPP1* appears to lie downstream of a number of signalling pathways with potential relevance to disease. We have demonstrated that uridine breakdown is promoted by MITF, a transcription factor associated with melanoma progression, which we show binds upstream of *UPP1* to promote its expression (Extended Data Fig. 7). In an accompanying study, Nwosu, Ward et al., demonstrate that UPP1 expression is under the control of KRAS–MAPK signalling^33^. It is notable that both MITF and NF-kB can act downstream of KRAS–MAPK^34–38^ and that some pancreatic cell lines with high uridine phosphorylase activity highlighted by Nwosu, Ward et al.^33^ unpublished data, also scored in our PRISM screen (Supplementary Table 1).

Finally, we found that RNA in the medium can replace glucose to promote cellular proliferation (Fig. 1i and Extended Data Fig. 6e). RNA is a highly abundant molecule, ranging from 4% of the dry weight of a mammalian cell to 20% of a bacterium^39^. Recycling of ribosomes through ribophagy, for example, plays an important role in supporting viability during starvation^40^. Cells of our immune system also ingest large quantities of RNA during phagocytosis, and we experimentally showed that the expression of *UPP1* increases with macrophage activation (Fig. 3i–k), including when cells are stimulated with RNA itself, suggesting the existence of a positive feedback loop. Uridine seems to be the only constituent of RNA that can be efficiently used for energy production, at least in K562 cells (Fig. 1h). Whereas the salvage of RNA to provide building blocks during starvation has long been appreciated for nucleotide synthesis, to our knowledge, its contribution to energy metabolism has not been considered in the past, except for some fungi that can grow on minimum media with RNA as their sole carbon source^41^. We speculate that, similar to glycogen and starch, RNA itself may constitute as large stock of energy in the form of a polymer, and that it may be used for energy storage and to support cellular function during starvation, or during processes associated with high energy costs such as the immune response.

## Methods

### Cell lines

K562 (CCL-243), 293T (CRL-3216), HeLa (CCL-2), A375 (CRL-1619), A2058 (CRL-11147), SH4 (CRL-7724), MDA-MB-435S (HTB-129), SK-MEL-5 (HTB-70), SK-MEL-30 (HTB-63) and THP1 (TIB-202) cell lines were obtained from the American Type Culture Collection (ATCC). UACC-62, UACC-257 and LOX-IMVI cells were obtained from the Frederick Cancer Division of Cancer Treatment and Diagnosis (DCTD) Tumor Cell Line Repository. All cell lines were re-authenticated by STR profiling at ATCC before submission of the manuscript and compared to ATCC and Cellosaurus (ExPASy) STR profiles in 2020, with the exception of THP1 (TIB-202) and U937 (CRL-1593.2), which were purchased from ATCC for the experiments. Cells lines from the PRISM collection were obtained from The PRISM Lab (Broad Institute) and were not further re-authenticated. MDA-MB-435S cells were previously assumed to be ductal carcinoma cells and recent gene expression analysis assigned them to the melanoma lineage (ATCC).

### Cell culture and cell growth assays

Cell line stocks were routinely maintained in DMEM (HeLa, 293T, K562, A375, A2058, SK-MEL-5, MDA-MB-435S) containing 1 mM sodium pyruvate (Thermo Fisher Scientific) with 25 mM glucose, 10% FBS (Thermo Fisher Scientific), 50 μg ml^−1^ uridine (Sigma), 4 mM l-glutamine and 100 U ml^−1^ penicillin–streptomycin (Thermo Fisher Scientific); or in RPMI (SH4, UACC-62, UACC-257, SK-MEL-30, LOX-IMVI, THP1) with 11.1 mM glucose, 10% FBS (Thermo Fisher Scientific), 2 mM l-glutamine and 100 U ml^−1^ penicillin–streptomycin (Thermo Fisher Scientific) under 5% CO_2_ at 37 °C. All growth assays, metabolomics, screens and bioenergetics experiments were performed in medium containing dialysed FBS. For growth experiments, an equal number of cells was counted, washed in PBS and resuspended in no-glucose DMEM (Thermo Fisher Scientific), or no-glucose RPMI (Teknova) complemented with 10% dialysed FBS (Thermo Fisher Scientific), 100 U ml^−1^ penicillin–streptomycin (Thermo Fisher Scientific) and 5–10 mM of glucose, galactose, uridine or mannose (all from Sigma) dissolved in water, or with an equal volume of water alone. For RNA and other nucleoside complementation assays, 0.5 mg ml^−1^ purified RNA from *Torula* yeast (Sigma) or the selected nucleosides (Sigma) were weighted and directly resuspend in DMEM. In all cases, cells were counted with a Vi-Cell Counter (Beckman) after 3 to 5 d of growth and only live cells were considered. Cell viability in glucose and galactose was determined using the same Vi-Cell Counter assay. Measurements were taken from distinct samples.

### Open reading frame screen

For ORF screening, K562 cells were infected with a lentiviral-carried ORFeome v8.1 library^2^ (Genome Perturbation Platform, Broad Institute) containing 17,255 ORFs mapping to 12,548 genes, in duplicate. Cells were infected at a multiplicity of infection of 0.3 and at 500 cells per ORF in the presence of 10 μg ml^−1^ polybrene (Millipore). After 72 h, cells were transferred to culture medium containing 2 μg ml^−1^ puromycin (Thermo Fisher Scientific) and incubated for an additional 48 h. On the day of the screen, cells were plated in screening medium containing no-glucose DMEM supplemented with 10% dialysed FBS, 1 mM sodium pyruvate (Thermo Fisher Scientific), 50 μg ml^−1^ uridine (Sigma) and 100 U ml^−1^ penicillin–streptomycin (Thermo Fisher Scientific) and 25 mM of either glucose or galactose (Sigma) at a concentration of 10^5^ cells per ml and with 500 cells per ORF. Cells were passaged every 3 d and 500 cells per ORF were harvested after 0, 9 and 21 d of growth. Total genomic DNA was isolated from cells using a NucleoSpin Blood kit (Clontech) using the manufacturer’s recommendations. Barcode sequencing, mapping and read count were performed by the Genome Perturbation Platform (Broad Institute). For screen analysis, log_2_ (normalized read counts) were used, and *P* values were calculated using a two-sided *t*-test. The presence of lentiviral recombination within the ORFeome library was not tested and as such genes that dropped out should be considered with caution, as these may represent unnatural proteins^42^.

### Stable gene over-expression

cDNAs corresponding to *GFP*, *UPP1*-FLAG and *UPP2-*FLAG were cloned in pWPI/Neo (Addgene). Lentiviruses were produced according to Addgene’s protocol. Twenty-four hours after infection, cells were selected with 0.5 mg ml^−1^ G418 (Thermo Fisher Scientific) for 48 h.

### Polyacrylamide gel electrophoresis and immunoblotting

Cells grown in routine medium were harvested, washed in PBS and lysed for 5 min on ice in RIPA buffer (25 mM Tris pH 7.5, 150 mM NaCl, 0.1% SDS, 0.1% sodium deoxycholate, 1% NP40 analogue, 1 × protease (Cell Signaling) and a 1:500 dilution of Universal Nuclease (Thermo Fisher Scientific)). Protein concentration was determined from total cell lysates using a DC protein assay (Bio-Rad). Gel electrophoresis was done on Novex Tris-Glycine gels (Thermo Fisher Scientific) before transfer using the Trans-Blot Turbo blotting system and nitrocellulose membranes (Bio-Rad). All immunoblotting was performed in Intercept Protein blocking buffer (Li-cor) or in 5% milk powder in TBST (TBS + 0.1% Tween-20). Washes were done in TBST. Specific primary antibodies were diluted at a concentration of 1:100–1:5,000 in blocking buffer. Fluorescent-coupled secondary antibodies were diluted at a ratio of 1:10,000 in blocking buffer. Membranes were imaged with an Odyssey CLx analyzer (Li-cor with Image Studio Lite v4.0) or by chemiluminescence. The following antibodies were used: FLAG M2 (Sigma, F1804), Actin (Abcam, ab8227), TUBB (Thermo, MA5-16308), UPP1 (Sigma, SAB1402388), MITF (Sigma, HPA003259), TYR (Santa Cruz sc-20035), MLANA (CST, 64718), HK2 (CST, 28675), GPI (CST, 94068), ALDOA (CST, 8060), TKT (CST, 64414), RPE (Proteintech, 12168-2-AP), PGM2 (Proteintech, 11022-1-AP), UCK2 (Proteintech, 10511-1-AP), TYMS (Proteintech, 15047-1-AP), S6 ribosomal protein (Santa Cruz, sc-74459) and phosphor-S6 (Santa Cruz, sc-293144). Two commercially available antibodies to UPP2 were tested (Sigma, SAB4301661; Abcam, ab153861), but no specific band could be detected.

### PRISM screen

A six-well plate containing a mixture of 482 barcoded adherent cancer cell lines (PR500)^13^ grown on RPMI (Life Technologies, 11835055) containing 10% FBS was prepared by The PRISM Lab (Broad Institute) seeded at a density of 200 cells per cell line. On day 0, the culture medium was replaced with no-glucose RPMI medium (Life Technologies, 11879020) containing 10% dialysed FBS and 100 U ml^−1^ penicillin–streptomycin and supplemented with 10 mM of either glucose or uridine (*n* = 3 replicate wells each). The medium was replaced with fresh medium on days 3 and 5. On day 6, all wells reached confluency and cells were lysed. Lysates were denatured, (95 °C) and total DNA from all replicate wells was PCR amplified using KAPA polymerase and primers containing Illumina flow cell-binding sequences. PCR products were confirmed to show single-band amplification using gel electrophoresis, pooled, purified using the Xymo Select-a-Size DNA Clean & Concentration kit, quantified using a Qubit 3 Fluorometer, and then sequenced via HiSeq (50 cycles, single read, library concentration of 10 pM with 25% PhiX spike-in) as previously described^43^. Barcode abundance was determined from sequencing, and unexpectedly low counts (for example, from sequencing noise) were filtered out from individual replicates so as not to unintentionally depress cell line counts in the collapsed data. Replicates were then mean-collapsed, and log fold change and growth rate metrics were calculated according to equations (1) and (2):

1 $$\log _2\,\mathrm{fold}\,\mathrm{change} = \log _2\left( {n_u/n_g} \right)$$

2 $$\mathrm{Growth}\,\mathrm{rate} = \frac{{\mathop {{\log }}\nolimits_2 \left( {n_f/n_0} \right)}}{t}$$

where *n*_u_ and *n*_g_ are counts from the uridine and glucose supplemented conditions, respectively, *n*_0_ and *n*_f_ are counts from the initial and final timepoints, respectively, and *t* is the assay length in days. Data analysis and correlation analysis were performed by The PRISM Lab following a published workflow^13^.

### RNA extraction, reverse transcription and qPCR

qPCR was performed using the TaqMan assays (Thermo Fisher Scientific). RNA was extracted from total cells grown in routine media with a RNeasy kit (Qiagen) and digested with DNase I before murine leukaemia virus reverse transcription using random primers (Promega) and a CFx96 qPCR machine (Bio-Rad) using the following TaqMan assays: Hs01066247_m1 (human *UPP1*), Mm00447676_m1 (mouse *Upp1*), Mm01331071_m1 (mouse *Upp2*), Hs01117294_m1 (human *MITF*), Hs01075618_m1 (human *UCK1*), Hs00989900_m1 (human *UCK2*), Mm00550050_m1 (mouse *Hmgcs2*), Hs00427620_m1 (human *TBP*) and Mm00782638_s1 (mouse *Rplp2*), Mm00434228_m1 (mouse Il-1B) and Mm01277042_m1 (mouse Tbp). An assay for human *UPP2* (Hs00542789_m1) was tested but no amplification could be detected. Human PBMCs and mouse BMDM data were normalized to *TBP*, and liver mouse data were normalized to *Rplp2*, both using the ΔΔCt method. qPCR primers for ChIP are described below.

### Chromatin immunoprecipitation

MDA-MB-435S cells were washed once with PBS and fixed with 1% formaldehyde in PBS for 15 min at room temperature. Fixation was stopped by adding glycine (final concentration of 0.2 M) for 5 min at room temperature. Cells were harvested by scraping with ice-cold PBS. Cell pellets were resuspended in SDS lysis buffer (50 mM Tris-HCl, pH 8.1, 10 mM EDTA, 1% SDS, protease inhibitor (Pierce Protease Inhibitor, EDTA-Free (Thermo Fisher Scientific))), incubated for 10 min at 4 °C, and sonicated to generate DNA fragments (around 500 base pairs) with a Qsonica Q800R2 system. Samples were centrifuged to remove debris and diluted tenfold in immunoprecipitation dilution buffer (16.7 mM Tris-HCl, pH 8.1, 1.2 mM EDTA, 0.01% SDS, 1.1% Triton-X100, 167 mM NaCl, protease inhibitor).

Chromatin (~50 μg) was pre-cleared with normal rabbit IgG (EMD Millipore) and protein A/G beads (Protein A/G UltraLink Resin (Thermo Fisher Scientific)) in low-salt buffer (20 mM Tris-HCl, pH 8.1, 2 mM EDTA, 0.1% SDS, 150 mM NaCl, protease inhibitor) containing 0.25 mg ml^−1^salmon sperm DNA and 0.25 mg ml^−1^ BSA for 2 h at 4 °C. Pre-cleared chromatin was incubated with 5 μl of anti-MITF (D5G7V (Cell Signaling Technology)) or 5 μg of normal rabbit IgG overnight at 4 °C (~1:10 vol:weight dilution). Samples were incubated with protein A/G beads for another 2 h at 4 °C. Immune complexes were washed sequentially twice with low-salt buffer, twice with high-salt buffer (20 mM Tris-HCl, pH 8.1, 2 mM EDTA, 0.1% SDS, 500 mM NaCl, protease inhibitor), LiCl buffer (250 mM LiCl, 1% NP40, 1% sodium deoxycholate, 1 mM EDTA, 10 mM Tris-HCl, pH 8.1, protease inhibitor) and twice with Tris-EDTA. After washes, immune complexes were eluted from beads twice with elution buffer (1% SDS, 10 mM dithiothreitol, 0.1 M NaHCO_3_) for 15 min at room temperature. Samples were de-crosslinked by overnight incubation at 65 °C and treated with proteinase K (Qiagen) for 1 h at 56 °C. DNA was purified with QIAquick PCR purification kit (Qiagen).

qPCR using KAPA SYBR FAST One-Step RT–qPCR Kit Universal (KAPA Biosystems) was performed to check MITF enrichments using the following primers: *UPP1*-TSS (5′-TGACCTTGGGTTAGTCCTAGA-3′) and (5′-AGCAGCCAGTTCTGTTACTC-3′); *UPP1*—3.5 kb (5′-AGCAACCTGGGAAAGTGATG-3′) and (5′-CGCCAACTCTCACTCATCATATAG-3′); *TYR* promoter (5′-GTGGGATACGAGCCAATTCGAAAG-3′) and (5′-TCCCACCTCCAGCATCAAACACTT-3′); *ACTB* gene body (5′-CATCCTCACCCTGAAGTACCC-3′) and (5′-TAGAAGGTGTGGTGCCAGATT-3′)

### Gene-specific CRISPR–Cas9 clone knockouts

To generate *UPP1*^KO^ single-cell clones in MDA-MB-435S and UACC-257 cells, a sgRNA targeting *UPP1* (TTGGATTTAAAAGTCTGACG) was ordered as complementary oligonucleotides (Integrated DNA Technologies) and cloned in pLentiCRISPRv2 (Addgene). Purified DNA was co-transfected with a GFP-expressing plasmid in the cell lines of interest using Lipofectamine 2000 (Thermo Fisher Scientific). After 48 h, cells were sorted using an MoFlo Astrios EQ Cell sorter and individual cells were seeded in a 96-well plate containing routine culture media for clone isolation. *UPP1* depletion in single-cell clones was assessed by protein immunoblotting using antibodies to UPP1. The region corresponding to the sgRNA targeting site in the *UPP1* gene was sequenced in MDA-MB-435S using TGGGAGCAACAGGGGTTAAG and TCAAGCATTTGTGGGTTGGTC primers and showed a homozygous 1-bp deletion in clone 1, heterozygous 4-bp and 9-bp deletions in clone 2, and heterozygous 1-bp and 2-bp insertions in clone 3. The 9-bp deletion in clone 2 is expected to produce a truncated protein (hypomorphic allele).

To deplete the expression of *ALDOA*, *GPI*, *HK2*, *PGM2*, *TKT*, *RPE*, *UCK1*, *UCK2* and *TYMS*, two sgRNAs were cloned into pLENTICRISPRv2. *UPP1*-expressing K562 cells were transduced with lentiviruses carrying these sgRNAs, selected with puromycin and the pooled population was analysed after 7–10 d. sgRNA sequences were: ALDOA_sg1 AATGGCGAGACTACCACCCA; ALDOA_sg2 AGGATGACACCCCCAATGCA; GPI_sg1

TGGGAGGACGCTACTCGCTG; GPI_sg2 TGACCCTCAACACCAACCAT; HK2_sg1 CATCAAGGAGAACAAAGGCG; HK2_sg2 TTACTTTCACCCAAAGCACA; PGM2_sg1

TGATTCTAGGAGCGTGAACA; PGM2_sg2 AATCCCCTGACTGATAAATG; TKT_sg1 GAAACAAGCTTTCACCGACG; TKT_sg2 CCTGCCCAGCTACAAAGTTG; RPE_sg1 ATATCTATCTGATTAGCCCA; RPE_sg2 CCCCAGAGTCTAGCATCCGG; UCK1_sg1 TGTGTCACAAAATCATAGGT; UCK1_sg2 CCGCTCACCCCTATCAGGAA; UCK2_sg1 TCTGCTCCGAGGTAAGGACA; UCK2_sg2 TACTGTCTATCCCGCAGACG; TYMS_sg1 TTCCAAGGGAGTGAAAATCT; TYMS_sg2 ATGTGCGCTTGGAATCCAAG.

### siRNA treatment

UACC-257 and MDA-MB-435S cells were transfected with a non-targeting siRNA (N-001206-14-05) or an siRNA targeting MITF (M-008674-0005; Dharmacon) using Lipofectamine RNAiMAX according to the manufacturer’s instruction. Cells were analysed 72 h after transfection and robust *MITF* knock-down was confirmed by qPCR.

### Immune cell isolation and differentiation

Human THP1 cultured cell lines were differentiated in routine medium containing 100 nM PMA (Sigma). After 2 d, the medium was changed for medium containing 100 ng ml^−1^ LPS (O111:B4, Sigma, L4391) or 1 mg ml^−1^ Torula yeast RNA (Sigma) and incubated for two additional days.

Mouse BMDMs were extracted from hips, femurs and tibias of three 13-week-old C57BL/6J male mice and plated in DMEM supplemented with 50 ng ml^−1^ M-CSF (ImmunoTools, 12343115), 10% heat-inactivated FBS, 1% penicillin–streptomycin and 1% HEPES. After 3 d, the medium was replenished with M-CSF-supplemented DMEM. On day 6, cells were detached, counted and replated at 2 × 10^6^ ml^−1^ per well of a six-well plate. Three hours after plating, cells were further treated with 0.1 µg µl^−1^ LPS O111:B4 (Sigma L4391), 1 mg ml^−1^ RNA (Sigma R6625) or 5 µg ml^−1^ R848 (Invivogen tlrl-r848) for 24 h. Cells treated with the IKK inhibitor BMS-345541 (Merck 401480) were pre-treated with 5 µM BMS-345541 for 1.5 h and then polarized with R848 and BMS-345541 for 24 h.

Human PBMCs were isolated from buffy coats of blood donors from a local transfusion centre. Buffy coats were centrifuged on a Lymphoprep (Stemcell, 07851) gradient followed by CD14^+^ purification with CD14 microbeads (Miltenyi, 130050201), according to manufacturer’s instruction. Isolated CD14^+^ cells were plated in RPMI medium supplemented with 50 ng ml^−1^ M-CSF (ImmunoTools, 11343113), 10% heat-inactivated FBS, 1% penicillin–streptomycin and 1% HEPES. After 3 d, the medium was replenished with M-CSF-supplemented DMEM. On day 6, cells were detached, counted and replated at 1.5–2 × 10^6^ ml^−1^ per well of a six-well plate. PBMC polarization was performed as with BMDMs.

### Genome-wide CRISPR–Cas9 screening

A secondary genome-wide CRISPR–Cas9 screening was performed using K562 cells expressing *UPP1*-FLAG and a lentiviral-carried Brunello library (Genome Perturbation Platform, Broad Institute) containing 76,441 sgRNAs^44^, in duplicate. Cells were infected with multiplicity of infection of 0.3 and at 500 cells per sgRNA in the presence of 10 μg ml^−1^ polybrene (Millipore). After 24 h, cells were transferred to culture medium containing 2 μg ml^−1^ puromycin (Thermo Fisher Scientific) and incubated for an additional 48 h. On day 7, the cells were plated in no-glucose DMEM containing 10% dialysed FBS and 100 U ml^−1^ penicillin–streptomycin and supplemented with 10 mM of either glucose or uridine at a concentration of 10^5^ cells per ml and with 1,000 cells per sgRNA. Cells were passaged every 3 d for 2 weeks and, on day 21, 1,000 cells per sgRNA were harvested. DNA isolation was performed as for the ORFeome screen.

CRISPR screen analysis was performed using a normalized *z*-score approach where raw sgRNA read counts were normalized to reads per million and then log_2_ transformed using the following formula: log_2_(reads from an individual sgRNA / total reads in the sample 10^6^ + 1)^45^. The log_2_ (fold change) of each sgRNA was determined relative to the pre-swap control. For each gene in each replicate, the mean log_2_ (fold change) in the abundance of all four sgRNAs was calculated. Genes with low expression (log_2_ (fragments per kilobase of transcript per million mapped reads) < 0) according to publicly available K562 RNA-sequencing data (sample GSM854403 in Gene Expression Omnibus series GSE34740) were removed. log_2_ (fold changes) were averaged by taking the mean across replicates. For each treatment, a null distribution was defined by the 3,726 genes with lowest expression. To score each gene within each treatment, its mean log_2_ (fold change) across replicates was *z*-score transformed, using the statistics of the null distribution defined as above.

### Metabolite profiling (steady state)

For steady-state metabolomics of glycolytic and PPP intermediates, an equal number of cells expressing *GFP* or *UPP1*-FLAG were washed in PBS and pre-incubated for 24 h in no-glucose DMEM supplemented with 10% dialysed FBS (Thermo Fisher Scientific), 100 U ml^−1^ penicillin–streptomycin (Thermo Fisher Scientific) and 5 mM of glucose, galactose or uridine (all from Sigma) dissolved in water, or with an equal volume of water alone. Cells were then re-counted and 2 × 10^6^ cells were seeded in fresh medium of the same formulation and incubated for two additional hours before metabolite extraction. Cells were pelleted and immediately extracted with 80% methanol, lyophilized and resuspended in 60% acetonitrile for intracellular LC–MS analysis.

### ^13^C_5_-uridine tracer on cultured cells

For tracer analysis on cultured cells, an equal number of cells expressing *GFP* or *UPP1*-FLAG were washed in PBS and pre-incubated in no-glucose DMEM or RPMI medium supplemented with 10% dialysed FBS (Thermo Fisher Scientific), 100 U ml^−1^ penicillin–streptomycin (Thermo Fisher Scientific) and 5 mM unlabelled uridine (all from Sigma) dissolved in water. After 24 h, the medium was changed for the same medium with the exception that ^13^C-labelled uridine ([1′,2′,3′,4′,5′-^13^C_5_] uridine, NUC-034, Omicron Biochemicals) was used. Cells were incubated for five additional hours before metabolite extraction. Cells were then harvested, the medium was removed and saved, and cellular pellets were resuspended in a 9:1 ratio (75% acetonitrile; 25% methanol:water) extraction mixture, spun at 20,000*g* for 10 min, and the supernatant was transferred to a glass sample vial for LC–MS analysis.

### Animal experiments

All animal experiments in this paper were approved by the Massachusetts General Hospital, the University of Massachusetts Institutional Animal Care and Use Committee, or the Swiss Cantonal authorities, and all relevant ethical regulations were followed. All animals used were male C57BL/6J mice purchased from The Jackson Laboratory, aged 8–13 weeks. All cages were provided with food and water ad libitum. Food and water were monitored daily and replenished as needed, and cages were changed weekly. A standard light–dark cycle of 12-h light exposure was used. Animals were housed at 2–5 per cage. The temperature was 21° ± 1 °C with 55% ± 10% humidity.

### ^13^C_5_-uridine tracer in mice

For in vivo tracing analysis, 8- to 12-week-old C57BL/6J male mice were fasted overnight or fed ad libitum and injected intraperitoneally with 0.2 M ^13^C-labelled uridine diluted in PBS to 0.4 g per kg body weight. After 30 min, blood and livers were collected from the mice under isoflurane anaesthesia. Liver was flash frozen in liquid nitrogen before subsequent analysis, and blood was collected in EDTA plasma tubes, spun and plasma was stored for further analysis. For plasma metabolite analysis, 117 μl of acetonitrile and 20 μl of LC–MS-grade water was added to 30 μl of plasma, the mixture was vortexed and left on ice for 10 min. The samples were then spun at 21,000*g* for 20 min, and 100 μl of the supernatant was transferred to a glass sample vial for downstream LC–MS analysis.

### Intracellular LC–MS analysis

For labelled and unlabelled LC–MS analysis of intracellular metabolites, 5 μl of sample was loaded on a ZIC-pHILIC column (Milipore). Buffer A was 20 mM ammonium carbonate, pH 9.6 and buffer B was acetonitrile. For each run, the total flow rate was 0.15 ml min^−1^ and the samples were loaded at 80% B. The gradient was held at 80% B for 0.5 min, then ramped to 20% B over the next 20 min, held at 20% B for 0.8 min, ramped to 80% B over 0.2 min, then held at 80% B for 7.5 min for re-equilibration. Mass spectra were continuously acquired on a Thermo Q-Exactive Plus run in polarity switching mode with a scan range of 70–1000 *m/z* and a resolving power of 70,000 (@200 *m/z*). Data were acquired using Xcalibur (v.4.1.31.9, Thermo Fisher). Data were analysed using TraceFinder (v.4.1, Thermo Fisher) and Progenesis (v.2.3.6275.47961) software, and labelled data were manually corrected for natural isotope abundance.

### Media/plasma LC–MS analysis

Media and plasma samples were subjected to the following LC–MS analysis: 10 μl of sample was loaded on a BEH Amide column (Waters). Buffer A was 20 mM ammonium acetate, 0.25% ammonium hydroxide, 5% acetonitrile, pH 9.0, while buffer B was acetonitrile. Samples were loaded on the column and the gradient began at 85% B, 0.22 ml min^−1^, held for 0.5 min, then ramped to 35% B over 8.5 min, then ramped to 2% B over 2 min, held for 1 min, then ramped to 85% B over 1.5 min and held for 1.1 min. The flow rate was then increased to 0.42 ml min^−1^ and held for 3 min for re-equilibration. Mass spectra were collected on a Thermo Q-Exactive Plus run in polarity switching mode with a scan range of 70–1,000 *m/z* and a resolving power of 70,000 (@200 *m/z*). Data were acquired using Xcalibur (v.4.1.31.9, Thermo Fisher). Data were analysed using TraceFinder (v.4.1, Thermo Fisher) and Progenesis (v.2.3.6275.47961) software, and labelled data were manually corrected for natural isotope abundance.

### Oxygen consumption and extracellular acidification rates by Seahorse XF analyzer

Approximately 1.25 × 10^5^ K562 cells were plated on a Seahorse plate in Seahorse XF DMEM medium (Agilent) supplemented with 10 mM glucose, galactose, mannose or uridine, or with an equal volume of water alone, and 4 mM glutamine (Thermo Fisher Scientific). FBS was omitted. Oxygen consumption and ECARs were simultaneously recorded by a Seahorse XFe96 analyzer (Agilent) using the Mito Stress Test protocol, in which cells were sequentially perturbed by 2 μM oligomycin, 1 μM CCCP and 0.5 μM antimycin (Sigma). Data were analysed using the Seahorse Wave Desktop Software (v.2.6.3, Agilent). Data were not corrected for carbonic acid derived from respiratory CO_2_.

### Lactate determination

Lactate secretion in the culture medium was determined using a glycolysis cell-based assay kit (Cayman Chemical). An equal number of K562 cells expressing *GFP* or *UPP1*-FLAG were washed in PBS and pre-incubated for 24 h in no-glucose DMEM medium supplemented with 10% dialysed FBS (Thermo Fisher Scientific), 100 U ml^−1^ penicillin–streptomycin (Thermo Fisher Scientific) and 5 mM glucose, galactose or uridine (all from Sigma) dissolved in water, or with an equal volume of water alone. Cells were then re-counted and seeded in fresh medium of the same formulation and incubated for three additional hours. Cells were then spun down and lactate concentration was determined on the supernatants (spent media).

### Gene Ontology analysis

Gene Ontology (GO) analysis was performed using GOrilla with default settings and using a ranked gene list as input^46^. Only GO terms constituted of < 500 genes and scoring at FDR < 0.001 with a minimum of two genes were considered significant and are displayed in the figures. The complete unfiltered data can be found in Supplementary Table 1.

### Gene-specific cDNA cloning and expression

cDNAs of interest were custom designed (Genewiz or IDT) and cloned into pWPI-Neo or pLV-lenti-puro using BamHI and SpeI (New England Biolabs).

### Statistics and reproducibility

All data are expressed as the mean ± s.e.m., with the exception of oxygraphic data that are expressed as the mean ± s.d. All reported sample sizes (*n*) represent biological replicate plates or a different mouse. All attempts at replication were successful. All Student’s *t*-tests were two sided. Statistical tests were performed using Microsoft Excel and GraphPad Prism 9.

### Reporting summary

Further information on research design is available in the Nature Portfolio Reporting Summary linked to this article.

### Supplementary information

Reporting Summary

Supplementary Table 1 Raw results and analysis of the ORFeome, CRISPR–Cas9 and PRISM screens.

### Source data

Source Data Fig. 1 Statistical source data.

Source Data Fig. 2 Statistical source data.

Source Data Fig. 3 Statistical source data.

Source Data Fig. 4 Statistical source data.

Source Data Extended Data Fig. 1 Statistical source data.

Source Data Extended Data Fig. 2 Statistical source data.

Source Data Extended Data Fig. 3 Statistical source data.

Source Data Extended Data Fig. 4 Statistical source data.

Source Data Extended Data Fig. 5 Statistical source data.

Source Data Extended Data Fig. 6 Statistical source data.

Source Data Extended Data Fig. 7 Statistical source data.

Source Data Extended Data Fig. 8 Statistical source data.

Source Data Extended Data Fig. 9 Statistical source data.

Source Data Extended Data Figs. 1, 3 and 6–8 Unprocessed western blots.

## Data Availability

All data generated or analysed during this study are included in the article and its Supplementary Information. Results of the ORFeome, the CRISPR–Cas9 and the PRISM screens are available in Supplementary Table 1. Data from the Cancer Cell Line Encyclopedia are available at https://depmap.org/portal/. Source data are provided with this paper.
