# Supplementary material for: Salvage of ribose from uridine or RNA supports glycolysis in nutrient-limited conditions
Source: Nat Metab. 2023 May 17;5(5):765–76. doi: 10.1038/s42255-023-00774-2 (PMC10229423; doi:10.1038/s42255-023-00774-2)
Supplement: Supplementary file 1 — Reporting Summary [file 42255_2023_774_MOESM1_ESM.pdf]

nature portfolio

Corresponding author(s): Alexis Jourdain and Vamsi Mootha

Last updated by author(s): 2023.02.09

Reporting Summary

Nature Portfolio wishes to improve the reproducibility of the work that we publish. This form provides structure for consistency and transparency in reporting. For further information on Nature Portfolio policies, see our Editorial Policies and the Editorial Policy Checklist.

Statistics

For all statistical analyses, confirm that the following items are present in the figure legend, table legend, main text, or Methods section.

n/a	Confirmed	
☐	☒	The exact sample size (*n*) for each experimental group/condition, given as a discrete number and unit of measurement
☐	☒	A statement on whether measurements were taken from distinct samples or whether the same sample was measured repeatedly
☐	☒	The statistical test(s) used AND whether they are one- or two-sided<br>*Only common tests should be described solely by name; describe more complex techniques in the Methods section.*
☒	☐	A description of all covariates tested
☒	☐	A description of any assumptions or corrections, such as tests of normality and adjustment for multiple comparisons
☐	☒	A full description of the statistical parameters including central tendency (e.g. means) or other basic estimates (e.g. regression coefficient) AND variation (e.g. standard deviation) or associated estimates of uncertainty (e.g. confidence intervals)
☒	☐	For null hypothesis testing, the test statistic (e.g. *F*, *t*, *r*) with confidence intervals, effect sizes, degrees of freedom and *P* value noted<br>*Give P values as exact values whenever suitable.*
☒	☐	For Bayesian analysis, information on the choice of priors and Markov chain Monte Carlo settings
☒	☐	For hierarchical and complex designs, identification of the appropriate level for tests and full reporting of outcomes
☐	☒	Estimates of effect sizes (e.g. Cohen's *d*, Pearson's *r*), indicating how they were calculated

Our web collection on statistics for biologists contains articles on many of the points above.

Software and code

Policy information about availability of computer code

Data collection	Xcalibur (v. 4.1.31.9, Thermofisher), Li-COR Odyssey, Seahorse
Data analysis	Microsoft Excel for Office 365 MSO, R (v 3.6.2), Tracefinder (v 4.1, Thermofisher) and Progenesis (2.3.6275.47961, nonlinear dynamics), Image Studio Lite (v4.0), Seahorse Wave (v2.6.3), GOrilla (http://cbl-gorilla.cs.technion.ac.il).

For manuscripts utilizing custom algorithms or software that are central to the research but not yet described in published literature, software must be made available to editors and reviewers. We strongly encourage code deposition in a community repository (e.g. GitHub). See the Nature Portfolio guidelines for submitting code & software for further information.

Data

Policy information about availability of data

All manuscripts must include a data availability statement. This statement should provide the following information, where applicable:

- Accession codes, unique identifiers, or web links for publicly available datasets
- A description of any restrictions on data availability
- For clinical datasets or third party data, please ensure that the statement adheres to our policy

All data generated or analyzed during this study are included in this published article (and its supplementary information files). Results of the ORFeome, the CRISPR/Cas9 and the PRISM screens are available in the Supplementary Table. Data from the Cancer Cell Line Encyclopedia is available at https://depmap.org/portal.

Human research participants

Policy information about [studies involving human research participants and Sex and Gender in Research.]

Reporting on sex and gender	*Use the terms sex (biological attribute) and gender (shaped by social and cultural circumstances) carefully in order to avoid confusing both terms. Indicate if findings apply to only one sex or gender; describe whether sex and gender were considered in study design whether sex and/or gender was determined based on self-reporting or assigned and methods used. Provide in the source data disaggregated sex and gender data where this information has been collected, and consent has been obtained for sharing of individual-level data; provide overall numbers in this Reporting Summary. Please state if this information has not been collected. Report sex- and gender-based analyses where performed, justify reasons for lack of sex- and gender-based analysis.*
Population characteristics	*Describe the covariate-relevant population characteristics of the human research participants (e.g. age, genotypic information, past and current diagnosis and treatment categories). If you filled out the behavioural & social sciences study design questions and have nothing to add here, write "See above."*
Recruitment	*Describe how participants were recruited. Outline any potential self-selection bias or other biases that may be present and how these are likely to impact results.*
Ethics oversight	*Identify the organization(s) that approved the study protocol.*

Note that full information on the approval of the study protocol must also be provided in the manuscript.

Field-specific reporting

Please select the one below that is the best fit for your research. If you are not sure, read the appropriate sections before making your selection.

☒ Life sciences ☐ Behavioural & social sciences ☐ Ecological, evolutionary & environmental sciences

For a reference copy of the document with all sections, see nature.com/documents/nr-reporting-summary-flat.pdf

Life sciences study design

All studies must disclose on these points even when the disclosure is negative.

Sample size	Statistical tests were not used to predefine sample size. For cell growth assays and metabolomics sample sizes of 3-4 were chosen to demonstrate moderate differences in commonly measured metabolic parameters, and because these are typical sample sizes reported for such experiments in the literature (see for example Shi et al., Nature Communications 2022). ORFeome and CRISPR/Cas9 screens were performed in biological duplicate, a common number of replicates for large-scale screens (see for example Yang et al., Nature Methods 2011 ; Doench et al., Nature Biotechnologies 2016). The PRISM screen was performed in biological triplicate, a common number of replicates for PRISM screens (see for example Corsello et al., Nature Cancer 2020.)
Data exclusions	No sample was excluded.
Replication	All attempts at replication were successful. All data points represent either experiments performed on an individual mouse (for in vivo data) or on different plates.
Randomization	Mass spectrometry runs involved randomization of sample order. Randomization was not relevant in the other experiments that were all performed in vitro.
Blinding	Blinding was not relevant for the experiments since all were performed with cell lines or indistinguishable animals.

Reporting for specific materials, systems and methods

We require information from authors about some types of materials, experimental systems and methods used in many studies. Here, indicate whether each material, system or method listed is relevant to your study. If you are not sure if a list item applies to your research, read the appropriate section before selecting a response.

Materials & experimental systems

n/a	Involved in the study
☐	☒ Antibodies
☐	☒ Eukaryotic cell lines
☒	☐ Palaeontology and archaeology
☐	☒ Animals and other organisms
☒	☐ Clinical data
☒	☐ Dual use research of concern

Methods

n/a	Involved in the study
☒	☐ ChIP-seq
☒	☐ Flow cytometry
☒	☐ MRI-based neuroimaging

Antibodies

Antibodies used

The following antibodies were used: FLAG M2 (Sigma, F1804), Actin (Abcam, ab8227), TUBB (Thermo, MA5-16308), UPP1 (Sigma, SAB1402388) and MITF (Sigma, HPA003259), TYR (Santa Cruz sc-20035), MLANA (CST, 64718), HK2 (CST, 28675), GPI (CST, 94068), ALDOA (CST, 8060), TKT (CST, 64414), RPE (Proteintech, 12168-2-AP), PGM2 (Proteintech, 11022-1-AP), UCK2 (Proteintech, 10511-1-AP), TYMS (Proteintech, 15047-1-AP), MITF antibody (D5G7V (Cell Signaling Technology)), S6 ribosomal protein (Santa Cruz, sc-74459), phosphor-S6 (Santa Cruz, sc-293144). Two commercially-available antibodies to UPP2 were tested (Sigma, SAB4301661 and Abcam, ab153861), but not specific band could be detected in immunoblotting.

Validation

All antibodies were raised against human proteins or peptides. HK2, GPI, ALDOA, TKT, RPE, PGM2, UCK2, UPP1 and TYMS antibodies were validated by targeted gene knock-out. FLAG and MITF antibody were validated by over-expressed of a FLAG-tagged protein. TYR was validated by over-expression of the protein by the supplier. MLANA was validated by immunodepletion by the supplier. MITF D5G7V, S6 ribosomal protein, TUBB and Actin were validated by the supplier (immunoblotting). Phosphor-S6 was validated by phosphatase treatment and immunoblotting by the supplier.

Eukaryotic cell lines

Policy information about cell lines and Sex and Gender in Research

Cell line source(s)

K562 (CCL-243), 293T (CRL-3216), HeLa (CCL-2), A375 (CRL-1619), A2058 (CRL-11147), SH4 (CRL-7724), MDA-MB-435S (HTB-129), SK-MEL-5 (HTB-70), SK-MEL-30 (HTB-63), U937 (CRL-1593.2) and THP1 (TIB-202) were obtained from ATCC. UACC-62, UACC-257 and LOX-IMVI were obtained from the Frederick Cancer Division of Cancer Treatment and Diagnosis.

Cells from the PRISM library (AGS_STOMACH, KYSE510_OESOPHAGUS, SNB75_CENTRAL_NERVOUS_SYSTEM, KP2_PANCREAS, SNU1214_UPPER_AERODIGESTIVE_TRACT, OVISE_OVARY, KYSE180_OESOPHAGUS, NCIH747_LARGE_INTESTINE, LN18_CENTRAL_NERVOUS_SYSTEM, JMSU1_URINARY_TRACT, UBLC1_URINARY_TRACT, SNU308_BILIARY_TRACT, SKNAS_AUTONOMIC_GANGLIA, NCIH1299_LUNG, SNU886_LIVER, MELJUSO_SKIN, KYSE30_OESOPHAGUS, TM87_SOFT_TISSUE, CAL29_URINARY_TRACT, WM2664_SKIN, GAMG_CENTRAL_NERVOUS_SYSTEM, RKN_SOFT_TISSUE, HSC2_UPPER_AERODIGESTIVE_TRACT, HEP3B217_LIVER, OVK18_OVARY, LI7_LIVER, ACCMESO1_PLEURA, 22RV1_PROSTATE, SW48_LARGE_INTESTINE, OE21_OESOPHAGUS, MSTO211H_PLEURA, JIMT1_BREAST, NCIH23_LUNG, SF295_CENTRAL_NERVOUS_SYSTEM, CFPAC1_PANCREAS, HEC265_ENDOMETRIUM, OSRC2_KIDNEY, TM31_CENTRAL_NERVOUS_SYSTEM, HT1376_URINARY_TRACT, 639V_URINARY_TRACT, SCABER_URINARY_TRACT, RH30_SOFT_TISSUE, SW1271_LUNG, DETROIT562_UPPER_AERODIGESTIVE_TRACT, RERFLCAD2_LUNG, SW1573_LUNG, PECAPJ15_UPPER_AERODIGESTIVE_TRACT, SCC25_UPPER_AERODIGESTIVE_TRACT, HCC1438_LUNG, A172_CENTRAL_NERVOUS_SYSTEM, MELHO_SKIN, LK2_LUNG, YKG1_CENTRAL_NERVOUS_SYSTEM, VMCUB1_URINARY_TRACT, TE5_OESOPHAGUS, CAOV3_OVARY, HOS_BONE, SKHEP1_LIVER, GOS3_CENTRAL_NERVOUS_SYSTEM, CAL54_KIDNEY, SNUC4_LARGE_INTESTINE, SF539_CENTRAL_NERVOUS_SYSTEM, NCIH2347_LUNG, HCC1195_LUNG, IPC298_SKIN, HSC3_UPPER_AERODIGESTIVE_TRACT, LC1SQSF_LUNG, HCC56_LARGE_INTESTINE, CORL105_LUNG, DANG_PANCREAS, EFO27_OVARY, CAMA1_BREAST, PC3_PROSTATE, OC314_OVARY, ISHIKAWAHERAKLIO02ER_ENDOMETRIUM, BXPC3_PANCREAS, SNU1066_UPPER_AERODIGESTIVE_TRACT, LMSU_STOMACH, NCIH841_LUNG, MDAMB436_BREAST, CL34_LARGE_INTESTINE, BCPAP_THYROID, TE4_OESOPHAGUS, SNU1076_UPPER_AERODIGESTIVE_TRACT, YH13_CENTRAL_NERVOUS_SYSTEM, NCIH460_LUNG, HT55_LARGE_INTESTINE, HUH28_BILIARY_TRACT, SH10TC_STOMACH, H4_CENTRAL_NERVOUS_SYSTEM, NCIH650_LUNG, PANC1005_PANCREAS, KALS1_CENTRAL_NERVOUS_SYSTEM, SNU1041_UPPER_AERODIGESTIVE_TRACT, DAOY_CENTRAL_NERVOUS_SYSTEM, COLO792_SKIN, S117_THYROID, T24_URINARY_TRACT, BICR6_UPPER_AERODIGESTIVE_TRACT, HA1E_KIDNEY, DU145_PROSTATE, SW1353_BONE, NCIH2452_PLEURA, SNU1105_CENTRAL_NERVOUS_SYSTEM, SJSA1_BONE, BC3C_URINARY_TRACT, MKN1_STOMACH, TE1_OESOPHAGUS, 2313287_STOMACH, SW620_LARGE_INTESTINE, CAPAN2_PANCREAS, HDQP1_BREAST, MON_SOFT_TISSUE, TCCPAN2_PANCREAS, NCIH292_LUNG, ISTMES1_PLEURA, NCIH1437_LUNG, SKMEL30_SKIN, SW1088_CENTRAL_NERVOUS_SYSTEM, LS411N_LARGE_INTESTINE, A2780_OVARY, OE33_OESOPHAGUS, SNU449_LIVER, VMRCRCW_KIDNEY, NCIH358_LUNG, SNU840_OVARY, HCC1143_BREAST, JHH4_LIVER, SKMES1_LUNG, ISTMES2_PLEURA, SW900_LUNG, TE10_OESOPHAGUS, RL952_ENDOMETRIUM, RERFLCMS_LUNG, MDAMB231_BREAST, KNS81_CENTRAL_NERVOUS_SYSTEM, CORL23_LUNG, TOV112D_OVARY, YD38_UPPER_AERODIGESTIVE_TRACT, GI1_CENTRAL_NERVOUS_SYSTEM, PANC0327_PANCREAS, NCIH596_LUNG, SBC5_LUNG, IGR37_SKIN, KMBC2_URINARY_TRACT, 253J_URINARY_TRACT, COV434_OVARY, EFO21_OVARY, SNU213_PANCREAS, COLO800_SKIN, BT549_BREAST, SNU869_BILIARY_TRACT, MFE319_ENDOMETRIUM, RERFLCAD1_LUNG, DV90_LUNG, TEN_ENDOMETRIUM, KNS62_LUNG, DBTRG05MG_CENTRAL_NERVOUS_SYSTEM, 8MGBA_CENTRAL_NERVOUS_SYSTEM, LCLC103H_LUNG, FADU_UPPER_AERODIGESTIVE_TRACT, HS944T_SKIN, PATU8988S_PANCREAS, SKMEL24_SKIN, TE8_OESOPHAGUS, IGR1_SKIN, UACC62_SKIN, NCIH446_LUNG, TE11_OESOPHAGUS, SW837_LARGE_INTESTINE, NCIH2126_LUNG, SNU61_LARGE_INTESTINE, HEC59_ENDOMETRIUM, KP4_PANCREAS, PC14_LUNG, IM95_STOMACH, CAL120_BREAST, RCM1_LARGE_INTESTINE, NCIH1838_LUNG,

PATU8902_PANCREAS, PANC0203_PANCREAS, SNU466_CENTRAL_NERVOUS_SYSTEM, BECKER_CENTRAL_NERVOUS_SYSTEM, SNU407_LARGE_INTESTINE, JHUEM2_ENDOMETRIUM, HCC1419_BREAST, MKN45_STOMACH, HT29_LARGE_INTESTINE, RD_SOFT_TISSUE, WM88_SKIN, HARA_LUNG, ECGI10_OESOPHAGUS, BICR16_UPPER_AERODIGESTIVE_TRACT, BFTC905_URINARY_TRACT, NCIH2170_LUNG, TUHR4TKB_KIDNEY, ASPC1_PANCREAS, NCIN87_STOMACH, OUMS23_LARGE_INTESTINE, NCIH226_LUNG, CAL12T_LUNG, NCIH520_LUNG, LUDLU1_LUNG, SNU245_BILIARY_TRACT, HCC1428_BREAST, CAL62_THYROID, RMGI_OVARY, NUGC3_STOMACH, KURAMOCHI_OVARY, HUPT3_PANCREAS, NCIH28_PLEURA, KP3_PANCREAS, DKMG_CENTRAL_NERVOUS_SYSTEM, OAW42_OVARY, EWS502_BONE, HUH7_LIVER, 769P_KIDNEY, SW1710_URINARY_TRACT, KYSE520_OESOPHAGUS, HT1080_SOFT_TISSUE, KMRC3_KIDNEY, PK45H_PANCREAS, 647V_URINARY_TRACT, TE6_OESOPHAGUS, NB1_AUTONOMIC_GANGLIA, L33_PANCREAS, NCIH2023_LUNG, SNU423_LIVER, SKUT1_SOFT_TISSUE, NCIH1373_LUNG, LOXIMVI_SKIN, CAKI1_KIDNEY, COLO668_LUNG, NCIH2009_LUNG, SNU601_STOMACH, NCIH1792_LUNG, KYSE70_OESOPHAGUS, SNU1077_ENDOMETRIUM, LU99_LUNG, MIAPACA2_PANCREAS, UACC257_SKIN, ABC1_LUNG, IGROV1_OVARY, COLO783_SKIN, QGP1_PANCREAS, NCIH1568_LUNG, SKMEL3_SKIN, CAL51_BREAST, HCT116_LARGE_INTESTINE, NCIH2052_PLEURA, BICR56_UPPER_AERODIGESTIVE_TRACT, MKN74_STOMACH, GCIY_STOMACH, HS294T_SKIN, PSN1_PANCREAS, TCCSUP_URINARY_TRACT, IALM_LUNG, NCIH1703_LUNG, TT_OESOPHAGUS, GB1_CENTRAL_NERVOUS_SYSTEM, ESS1_ENDOMETRIUM, HPAC_PANCREAS, A673_BONE, PANC0403_PANCREAS, HCC95_LUNG, HCC38_BREAST, MPP89_PLEURA, SIMA_AUTONOMIC_GANGLIA, NCIH1651_LUNG, SNU398_LIVER, NCIH1915_LUNG, TT2609C02_THYROID, TE14_OESOPHAGUS, PANC0813_PANCREAS, CJM_SKIN, EN_ENDOMETRIUM, FTC133_THYROID, TE9_OESOPHAGUS, KMRC20_KIDNEY, ACHN_KIDNEY, JHH1_LIVER, LNCAPCLONEFGC_PROSTATE, MHHES1_BONE, HCC1806_BREAST, RT4_URINARY_TRACT, KYSE140_OESOPHAGUS, 5637_URINARY_TRACT, A101D_SKIN, NCIH2444_LUNG, BHT101_THYROID, RERFLCKJ_LUNG, MFE280_ENDOMETRIUM, 8305C_THYROID, SNU668_STOMACH, CCFSTTG1_CENTRAL_NERVOUS_SYSTEM, HCC515_LUNG, NCIH1339_LUNG, NCIH1975_LUNG, PECAPJ41CLONED2_UPPER_AERODIGESTIVE_TRACT, 253JBV_URINARY_TRACT, HEC151_ENDOMETRIUM, HGC27_STOMACH, HCC827_LUNG, NCIH661_LUNG, MCF7_BREAST, MESSA_SOFT_TISSUE, SNU1079_BILIARY_TRACT, LN229_CENTRAL_NERVOUS_SYSTEM, HEC1A_ENDOMETRIUM, PK59_PANCREAS, SNU410_PANCREAS, HUCCT1_BILIARY_TRACT, GCT_SOFT_TISSUE, MDAMB468_BREAST, SW1990_PANCREAS, BICR31_UPPER_AERODIGESTIVE_TRACT, SNU761_LIVER, EBC1_LUNG, LOVO_LARGE_INTESTINE, MEWO_SKIN, WM793_SKIN, ZR751_BREAST, NCIH2077_LUNG, SNUC2A_LARGE_INTESTINE, SKMEL5_SKIN, NCIH1944_LUNG, BEN_LUNG, OV7_OVARY, HCC1937_BREAST, SNU719_STOMACH, 786O_KIDNEY, SNGM_ENDOMETRIUM, JHOC5_OVARY, NCIH522_LUNG, PATU8988T_PANCREAS, SKNEP1_BONE, OVCAR8_OVARY, NCIH838_LUNG, A549_LUNG, ONCODG1_OVARY, NCIH1623_LUNG, OE19_OESOPHAGUS, HEC1B_ENDOMETRIUM, HCC78_LUNG, MG63_BONE, MKN7_STOMACH, G402_SOFT_TISSUE, SH4_SKIN, SKOV3_OVARY, NUGC4_STOMACH, OC316_OVARY, RKO_LARGE_INTESTINE, JHH5_LIVER, LOUNH91_LUNG, OVTOKO_OVARY, TT_THYROID, T3M10_LUNG, LS1034_LARGE_INTESTINE, MDAMB435S_SKIN, HS729_SOFT_TISSUE, SW780_URINARY_TRACT, YD8_UPPER_AERODIGESTIVE_TRACT, PECAPJ49_UPPER_AERODIGESTIVE_TRACT, JHH6_LIVER, NCIH1581_LUNG, HS939T_SKIN, NCIH1793_LUNG, SKES1_BONE, PK1_PANCREAS, BT474_BREAST, SNU1196_BILIARY_TRACT, SW579_THYROID, SKNBE2_AUTONOMIC_GANGLIA, HCC1359_LUNG, T98G_CENTRAL_NERVOUS_SYSTEM, U118MG_CENTRAL_NERVOUS_SYSTEM, A375_SKIN, KU1919_URINARY_TRACT, HS766T_PANCREAS, HCC4006_LUNG, YAPC_PANCREAS, KMRC1_KIDNEY, CALU6_LUNG, WM983B_SKIN, RERFLCAI_LUNG, CHL1_SKIN, BFTC909_KIDNEY, SNU685_ENDOMETRIUM, AN3CA_ENDOMETRIUM, T47D_BREAST, JHOS2_OVARY, HCT15_LARGE_INTESTINE, NCIH2122_LUNG, U251MG_CENTRAL_NERVOUS_SYSTEM, CADOES1_BONE, HLF_LIVER, UO31_KIDNEY, J82_URINARY_TRACT, SW480_LARGE_INTESTINE, UMUC1_URINARY_TRACT, HCC15_LUNG, CALU1_LUNG, MCAS_OVARY, KYSE270_OESOPHAGUS, U2OS_BONE, HCC44_LUNG, COV362_OVARY, SKMEL2_SKIN, COLO679_SKIN, G292CLONEA141B1_BONE, NCIH727_LUNG, YD10B_UPPER_AERODIGESTIVE_TRACT, JHH7_LIVER, NCIH1648_LUNG, HEC6_ENDOMETRIUM, PANC1_PANCREAS, SNU81_LARGE_INTESTINE, NCIH2030_LUNG, LS513_LARGE_INTESTINE, KYSE150_OESOPHAGUS, NCIH1573_LUNG, NCIH2110_LUNG, HS852T_SKIN, HMC18_BREAST, NCIH2228_LUNG, NCIH1650_LUNG, HT144_SKIN, NCIH2172_LUNG, CAL78_BONE, KYSE410_OESOPHAGUS, NCIH2087_LUNG, ONS76_CENTRAL_NERVOUS_SYSTEM, 8505C_THYROID, HEC108_ENDOMETRIUM, NCIH1693_LUNG, YD15_SALIVARY_GLAND, COLO680N_OESOPHAGUS, 42MGBA_CENTRAL_NERVOUS_SYSTEM, FTC238_THYROID, COLO741_SKIN, JHOM1_OVARY, SF126_CENTRAL_NERVOUS_SYSTEM, RVH421_SKIN, RT112_URINARY_TRACT, PLCPRF5_LIVER, MFE296_ENDOMETRIUM, NCIH1355_LUNG, OV56_OVARY, GP2D_LARGE_INTESTINE, KNS60_CENTRAL_NERVOUS_SYSTEM, NCIH1048_LUNG, MALME3M_SKIN, MDAMB175VII_BREAST, NCIH1563_LUNG, CAL27_UPPER_AERODIGESTIVE_TRACT, TOV21G_OVARY, NCIH322_LUNG, RMUGS_OVARY, SUIT2_PANCREAS, HEC251_ENDOMETRIUM, HUH6_LIVER, KNS42_CENTRAL_NERVOUS_SYSTEM, G401_SOFT_TISSUE, EFM192A_BREAST, HEYA8_OVARY, RCC10RGB_KIDNEY, SQ1_LUNG, LXF289_LUNG, ES2_OVARY, A704_KIDNEY, LS180_LARGE_INTESTINE, KELLY_AUTONOMIC_GANGLIA, A2058_SKIN, K029AX_SKIN, NCIH1435_LUNG, SNU46_UPPER_AERODIGESTIVE_TRACT, VMRCRCZ_KIDNEY, DMS273_LUNG, SW948_LARGE_INTESTINE, WM1799_SKIN, SKLU1_LUNG, G361_SKIN) were obtained from the Broad-Novartis cancer cell line encyclopedia prior to PRISM barcoding and are described in Corsello et al., Nature Cancer 2020.

Authentication

All cell lines were re-authenticated by STR profiling at ATCC or Genetica prior submission of the manuscript and compared to ATCC and Cellosaurus (ExPASy) STR profiles, with the exception of THP1 and U937 that were acquired from ATCC for these experiments and were not re-identificated.

Mycoplasma contamination

All cell lines were tested for mycoplasma contamination bi-monthly which was negative.

Commonly misidentified lines
(See ICLAC register)

MDA-MB-435S are sometimes wrongly assumed to be breast cancer cells. Recent gene expression analysis (ATCC) re-assigned MDA-MB-435S to the melanoma lineage (ATCC) and here MDA-MB-435S were used as such.

Animals and other research organisms

Policy information about studies involving animals; ARRIVE guidelines recommended for reporting animal research, and Sex and Gender in Research

Laboratory animals	All animals used were male C57BL6/J mice purchased from Jackson labs, aged 8-13 weeks. All cages were provided with food and water ad-libitum. Food and water were monitored daily and replenished as needed, and cages were changed weekly. A standard light-dark cycle of –12h light exposure was used. Animals were housed 2–5 per cage. Temperature was 21° ± 1°C with 55% ± 10% humidity.
Wild animals	This study did not involve wild animals.
Reporting on sex	Sex was not considered in this study. Only male mice were used. The choice of male was random.
Field-collected samples	This study did not involve field-collected samples.
Ethics oversight	All animal protocols were approved by the MGH IACUC and the Swiss Cantonal authorities.

Note that full information on the approval of the study protocol must also be provided in the manuscript.
