# Supplementary figures and images for: Salvage of ribose from uridine or RNA supports glycolysis in nutrient-limited conditions
Source: Nat Metab. 2023 May 17;5(5):765–76. doi: 10.1038/s42255-023-00774-2 (PMC10229423; doi:10.1038/s42255-023-00774-2)

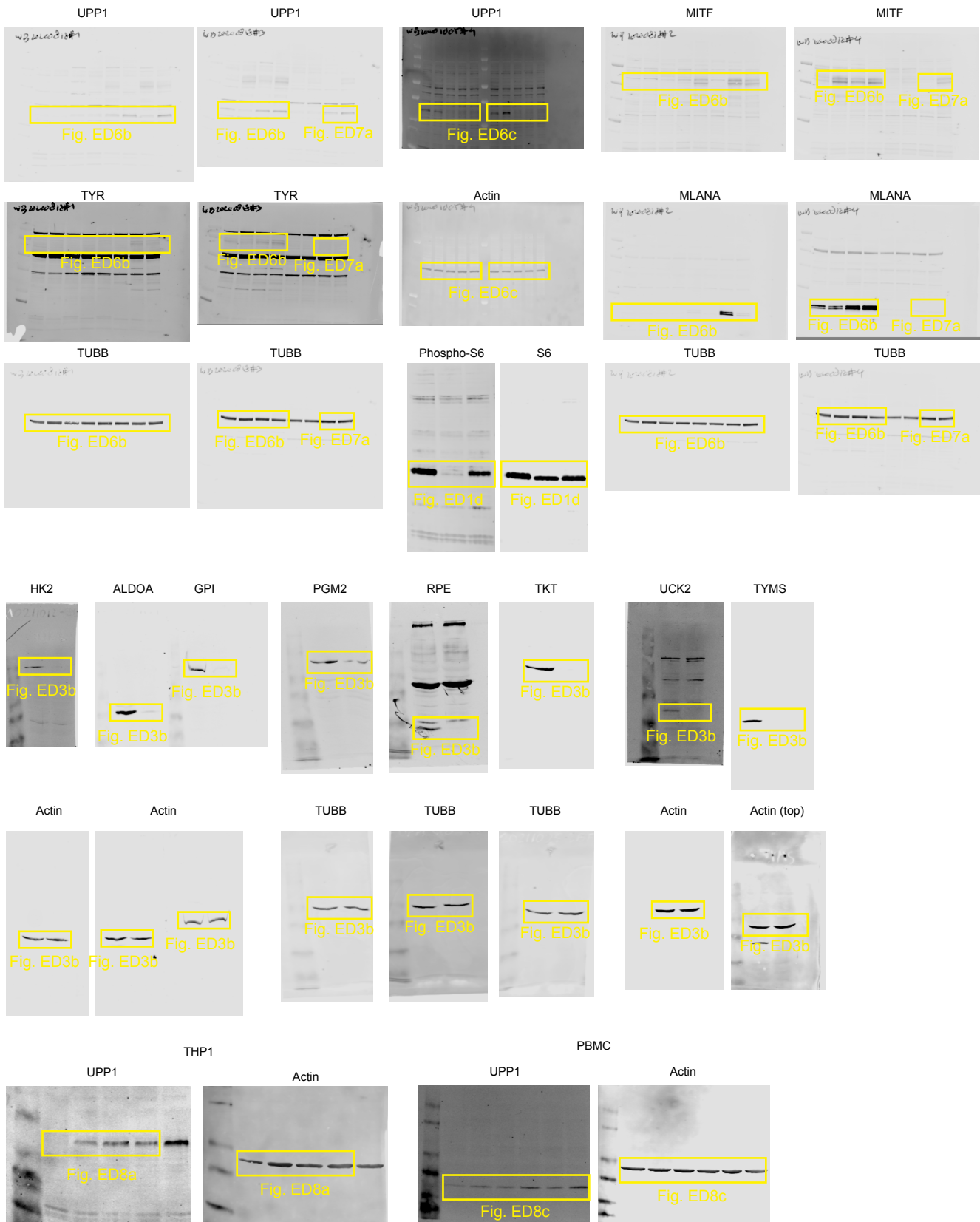

Supplement: Supplementary file 16 — Unprocessed western blots. [file 42255_2023_774_MOESM16_ESM.pdf]
